# Bacterial Resilience and Vulnerability to Neonicotinoid Seed Treatments in Soil: Short‐Term Community Responses

**DOI:** 10.1111/1758-2229.70339

**Published:** 2026-04-07

**Authors:** Sharmin Akter, Julia Jasonsmith, Nilantha R. Hulugalle, Craig L. Strong

**Affiliations:** ^1^ Fenner School of Environment and Society, College of Systems and Society Australian National University Canberra Australian Capital Territory Australia; ^2^ Soil Resource Development Institute Ministry of Agriculture Dhaka Bangladesh

**Keywords:** bacterial diversity, clothianidin, differential abundance analysis, imidacloprid, thiamethoxam, wheat microcosms

## Abstract

We explored the short‐term impacts of three neonicotinoids (imidacloprid, thiamethoxam, and clothianidin) on soil bacterial community composition and diversity in wheat‐planted microcosms. Neonicotinoids were applied as seed treatments, and soil samples were collected over 10 days. Overall alpha diversity metrics showed no significant treatment‐ or time‐dependent shifts; however, post hoc analyses revealed transient, treatment‐specific responses at individual sampling time points. Thiamethoxam and clothianidin significantly increased diversity and evenness at early time points, while imidacloprid reduced diversity by Day 10. Clothianidin was also associated with a short‐term increase in estimated species richness. Actinobacteriota and Proteobacteria dominated across treatments, with Firmicutes increasing and Bacteroidota declining with time. The minor phylum Methylomirabilota exhibited a significant treatment effect. Sampling day and day‐treatment interaction significantly influenced community structure. *Mesorhizobium* enriched under all neonicotinoids. Imidacloprid enhanced *Massilia* and suppressed Solirubrobacterales and Chloroflexia. Thiamethoxam enriched *Gaiella*, *Solirubrobacter*, and *Massilia* but suppressed *Nitrospira*. Clothianidin enriched *Solirubrobacter* and *Lysobacter* but suppressed *Methyloceanibacter* and *Nitrospira*. Haliangiaceae were positively correlated with sampling days, while Flavobacteriaceae and Microscillaceae were negatively correlated. Yersiniaceae and Solirubrobacteraceae were negatively correlated with imidacloprid and Mycobacteriaceae with thiamethoxam. These findings highlight the need for longer‐term and functional investigations into neonicotinoid impacts on soil microbial communities and ecosystem health.

## Graphical abstract

This study investigated short‐term impacts of seed treatment‐derived neonicotinoids on soil bacterial communities in wheat‐planted microcosms. Over 10 days, significant Day and Day × Treatment effects on bacterial community structure were observed, with selective enrichment and suppression of specific taxa despite limited overall changes in alpha diversity.

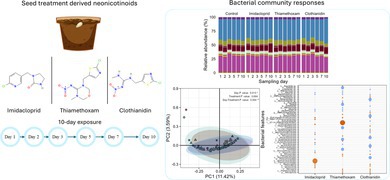

## 1 Introduction

Within the surface millimetre of the soil lies a diverse microscopic community that collectively functions to produce the complex web of interactions described as the soil ecosystem (Manda et al. 2023). This microscopic community or soil microbiome is a highly dynamic assemblage of bacteria, fungi, protozoa, archaea, and other microorganisms. The quality and health of soil are often characterised by the functional capacity of these microbes (Potts et al. 2023; Hou et al. 2020). They are collectively responsible for maintaining soil fertility and ecosystem resilience and drive key soil processes such as nutrient cycling, organic matter decomposition, and soil structure maintenance (Uzoh et al. 2021; Kästner and Miltner 2018; Dini‐Andreote and Van Elsas 2019). Among the soil microorganisms, bacteria are one of the most dominant, with their biomass often ranging from 100 to 10,000 times greater than that of other key microbial groups in the soil microbiome (Fierer 2017). Bacteria perform critical roles in several biochemical processes such as carbon mineralisation, nitrogen transformation and sulfur reduction and oxidation, with these processes fundamental to broader ecosystem health (Basu et al. 2021), organic matter decomposition, soil fertility, and nutrient availability (Yadav et al. 2021). Additionally, bacteria promote plant growth via biogeochemical processes such as fixing atmospheric nitrogen, solubilising phosphate through the secretion of organic acids, and releasing soluble potassium from otherwise insoluble rocks and silicate materials (Alori et al. 2017; Ribeiro et al. 2020; Raza et al. 2023; Sindhu et al. 2014). However, these crucial processes are susceptible to disturbances through human activities, particularly the widespread use of chemical inputs in modern farming practices (Yang et al. 2021; Martyniuk et al. 2019).

Modern agriculture practices impose a range of obvious and subtle stresses that can lead to instability and changes in the composition of soil microbial communities (Ni et al. 2025; Gupta et al. 2022). One such stress has been the introduction and rapid increase in the use of neonicotinoid insecticides, which are highly effective in protecting crops from a broad range of insect pests (Thompson et al. 2020; Thany 2023) and are used at different phases of cultivation and during post‐harvest storage (Liu et al. 2010; Orikpete et al. 2025). Neonicotinoids are a class of systemic insecticides characterised by nitroguanidine (—N—NO_2_) or cyanoamidine (=N—CN) functional groups, which confer high affinity for insect nicotinic acetylcholine receptors (nAChRs) (Buszewski et al. 2019). They are widely applied for the control of sap‐feeding pests such as aphids, leafhoppers, whiteflies, and thrips, as well as several soil‐dwelling coleopteran larvae (Jeschke and Nauen 2005; Rawal et al. 2025). They are especially controversial because their adverse effects on organisms at the bottom of the food chain are considered to have been inadequately studied (Bakker et al. 2020; Overton et al. 2021; Umina et al. 2019; Bonmatin et al. 2021). They can be applied in several ways—sprayed onto leaves, drenched into the soil, applied to seeds as treatment, or used as granules over various crops (Briceño et al. 2024). Their longevity in soil, high solubility in water, and weak adsorption properties enable neonicotinoids to persist and travel through soil and water systems (Bonmatin et al. 2015; Lamers et al. 2011; Li et al. 2018; Zhang et al. 2023).

Neonicotinoids' potential toxicity to non‐target soil microorganisms has been highlighted by several studies. Castillo‐Díaz et al. (2017) observed immediate significant reductions in bacterial abundance and shifts in community structure following imidacloprid application. Similarly, Wu et al. (2021) further noted that thiamethoxam had a short‐term impact on soil bacterial abundance, leading to a reduction in microbial diversity and changes in bacterial community structure. Li et al. (2018) reported that higher concentrations of clothianidin suppressed bacterial species richness, though overall community diversity was unaffected.

Neonicotinoids not only affect bacterial diversity and abundance but also disrupt key bacterial‐driven processes, particularly nutrient cycling and community interactions. Imidacloprid inhibited nitrifying bacteria, reducing nitrification rates and stimulating ammonification, while also suppressing critical processes like nitrogen transformation and carbon mineralisation (Mahapatra et al. 2017; Cycoń and Piotrowska‐Seget 2015). Yamaguchi et al. (2021) reported that dinotefuran promoted ammonia oxidation but suppressed nitrite oxidation, further disrupting nitrogen cycling.

In addition to changes in community structure and specific processes, neonicotinoids also induce broader metabolic and functional shifts within bacterial communities. Yu et al. (2020) found that dinotefuran and thiamethoxam altered carbon metabolism in bacterial communities and changed the genetic profiles of specific bacterial taxa, potentially leading to shifts in soil microbial community function. Neonicotinoid seed treatments selectively affected bacterial gene expression, particularly genes related to regulatory functions, metabolic activities, DNA repair and most notably, heat shock proteins, with these effects varying across and between growing seasons (Parizadeh et al. 2023). These metabolic changes reflected how neonicotinoid exposure can reshape bacterial communities, potentially leading to long‐term disruptions in soil ecosystems.

In Australian wheat ( *Triticum aestivum* L.) production, neonicotinoids play a key role in pest management (Overton et al. 2021; Umina et al. 2019). Neonicotinoid seed treatments provide effective protection for young wheat plants against aphids (Labrie et al. 2020; Miao et al. 2014), but their use within the soil raises concerns regarding their persistence and potential ecological impacts on soil microbial communities (Wettstein et al. 2016; Zheng et al. 2022). This study investigated the impact of three neonicotinoids, applied as seed treatments in wheat, on soil bacterial communities. Imidacloprid, thiamethoxam, and clothianidin were selected because they are among the most widely used neonicotinoid seed treatments in cereal production and represent closely related yet chemically distinct compounds with differing soil persistence and degradation profiles (Thompson et al. 2020). Seed treatment was selected to reflect common agricultural practice and realistic exposure pathways, where microbial communities are exposed to active ingredients via root exudates, seed leachates, and rhizosphere interactions. We hypothesised that (i) neonicotinoid seed treatment would alter the soil bacterial community composition and structure, leading to changes in diversity and taxa interactions, and (ii) these changes would vary over time, with each neonicotinoid causing distinct temporal patterns in community structure and taxa abundance shifts. To test these hypotheses, an experiment was conducted for 10 days in small pots under controlled laboratory conditions. Neonicotinoids were applied as a seed treatment to wheat seeds in separate microcosms. Soils associated with treated seeds were sampled to capture the immediate and temporal dynamics of bacterial responses. The short duration of the experiment was intentional, aimed not at chemical risk assessment but at capturing early‐stage microbial responses to neonicotinoid seed treatments.

## 2 Materials and Methods

### 2.1 Chemical and Seed Material

Analytical standards (≥ 98.0% HPLC area purity) of three neonicotinoid chemicals were purchased from Sigma‐Aldrich Pty Ltd. (A subsidiary of Merck), Australia, with these being imidacloprid (C_9_H_10_ClN_5_O_2_), thiamethoxam (C_8_H_10_ClN_5_O_3_S), and clothianidin (C_6_H_8_ClN_5_O_2_S). Wheat seeds were purchased locally in Canberra, Australia with no specific cultivar information available.

### 2.2 Soil Sampling and Preparation

Five kilograms of the A horizon from a Red Luvisol (Iuss Working Group Wrb 2015) soil with no pesticide application history was collected and transported back to the laboratory for analysis. The sampling location has a long history of being well‐mulched and shaded with minimal disturbance. The A horizon was ~0.2 m deep, dominated by < 10 mm aggregates and showed a high incidence of macroinvertebrate & earthworm activity.

The collected soil sample was carefully homogenised to create a composite soil sample. To ensure uniformity, the homogenised soil was sieved (≤ 2 mm) to remove roots, stones and earthworms. The soil had a sandy loam texture (70 g/100 g sand (0.05–2 mm), 14 g/100 g silt (0.02–0.05 mm), and 16 g/100 g clay (< 0.02 mm)). Its pH was 6.7, electrical conductivity (EC_1:5_) 0.41 dS/m, total organic matter 8.4 g/100 g, total carbon 4.8 g/100 g, total nitrogen 0.43 g/100 g, C/N 11.0, Bray‐1 extractable phosphorus 232 mg/kg, exchangeable Ca 18 cmol(+)/kg, exchangeable Mg 3.5 cmol(+)/kg, exchangeable K 1.4 cmol(+)/kg, exchangeable Na < 0.07 cmol(+)/kg, effective CEC 23 cmol(+)/kg, exchangeable sodium percentage (ESP) 0.16 and Ca/Mg 5.1. All methods used to analyse the soil are summarised in Table S1.

### 2.3 Seed Treatment and Experimental Setup

Wheat seeds were treated with analytical‐grade active ingredients to simulate the application of three commercial neonicotinoid formulations at field‐relevant rates. The target application rate for each compound was based on the recommended commercial dose of 120 mL of formulated product per 100 kg of seed, using products with the following active ingredient (a.i.) concentrations: 60% for imidacloprid (Bayer Cropscience Australia 2023), 60% for clothianidin (Bayer Cropscience 2023) and 35% for thiamethoxam (Syngenta Australia 2023). This corresponds to active ingredient application rates of 72 g a.i. per 100 kg seed for imidacloprid and clothianidin, and 42 g a.i. per 100 kg of seed for thiamethoxam. To replicate these rates for 20 g of seed used per treatment, the following amounts of analytical‐grade (99.7% purity) compounds were used: 14.4 mg of imidacloprid, 14.4 mg of clothianidin, and 8.4 mg of thiamethoxam. Each compound was weighed into a sterile 50 mL specimen jar inside a certified fume cupboard. 5 mL of Milli‐Q water was added to dissolve the compound, followed by the addition of 20 g of wheat seeds. The contents were gently agitated to ensure uniform aqueous coating. The treated seeds were left in sealed jars at room temperature for 1 h to allow adequate absorption. Seeds were used immediately after this treatment period without drying or storage.

Control seeds were handled using the same procedure but without the addition of insecticides. All treatments were carried out under appropriate laboratory safety protocols, including the use of gloves, lab coat, face shield, safety goggles and respiratory protection. All containers and tools were either sterilised between treatments or disposed of according to institutional biosafety guidelines.

We used 30 mL pots with lids, each containing 40 g of soil. Two small holes were made in the sides of each pot prior to filling them with soil and were temporarily sealed with adhesive tape. To standardise the starting conditions, we compacted the soil in each pot with a penetrometer set at a pressure of approximately 29,400 Pa. Soil properties that could potentially influence bacterial populations, such as temperature, pH, moisture, texture, and electrical conductivity (Akter et al. 2023) were identical in all pots.

Two treated or control seeds were planted in each pot. The soil was moistened to a gravimetric moisture content of 32 g/100 g using Milli‐Q water (deionised water) to maintain consistent moisture conditions across all pots. After watering, the pots were capped with lids and incubated in the dark at room temperature (22°C). No fertiliser was added.

Since time was a key experimental variable, soil samples were collected at six specific intervals: day 1 (pretreatment baseline, equivalent to time zero), 2 (24 h after treatment), 3, 5, 7, and 10, to observe temporal changes in bacterial communities. To minimise variability, soil samples were collected each day beginning at 9:30 AM, following a consistent sampling order across treatments. Each treatment and control group included five replications for each sampling day, resulting in a total of 120 pots arranged in a randomised complete block design.

On each sampling day, designated pots from each treatment group were withdrawn from the experiment for soil sampling. A clean micro spatula was pushed horizontally through the full diameter of the pot via the side hole to collect a soil core (~0.25 g). To prevent contamination, disposable nitrile gloves were used for each sample, and the spatula was sterilised between collections. Collected soil samples from the microcosms were immediately processed for subsequent DNA extraction to preserve microbial integrity and minimise any potential changes in the microbial community. After pot preparation, the entire experiment was carried out inside a fume cupboard to ensure safety and minimise contamination.

### 2.4 Extraction of Genomic DNA and Next‐Generation Amplicon Sequencing

Genomic DNA (gDNA) was extracted from the soil samples using the DNeasy PowerSoil Pro Kit (QIAGEN, Germany) on the same day of soil sample collection, following the manufacturer's protocol (Wu 2022). Subsequently, the concentrations of gDNA were determined using a Qubit 4 Fluorometer with the dsDNA BR Assay Kit for Qubit. The quality of the gDNA was assessed employing the NanoDrop Lite Spectrophotometer (Thermo Fisher Scientific, Wilmington, DE, USA), after which the extracted gDNA was securely stored at −20°C, awaiting downstream analysis.

The extracted gDNA samples were sequenced by the Ramaciotti Centre for Genomics (UNSW, Australia), targeting the 16S rRNA gene. The primer pairs 27f–519r for the V1–V3 regions of the 16S rRNA gene were used to amplify these regions (Lane et al. 1985; Lane 1991). Pair‐end targeted amplicon sequencing was performed on the MiSeq v3 platform using a two‐step PCR workflow (Illumina Inc., San Diego, CA, United States) with a read length of 2 × 300 bp. Detailed primer sequences are provided in Table S2.

### 2.5 Bioinformatics

The initial metagenomic data consisted of paired‐end FASTQ files generated by Illumina sequencing. These files had been demultiplexed by sample, with barcodes, Illumina adapters, and unique dual indices already trimmed during the de‐multiplexing process. Non‐biological nucleotides (primers) were removed from the demultiplexed FASTQ files using Cutadapt tool (v2.10) (Martin 2011) prior to processing. The sequencing data were processed following the version 1.8 DADA2 workflow, using the ‘dada2’ package (v1.32) (Callahan et al. 2016). The quality of control was performed using ‘plotQualityProfile’. Sequences were truncated to 275 bp for forward reads and 245 bp for reverse reads, with a maximum of 2 and 5 expected errors, respectively, based on the observed quality score distribution (QS > 20). Primer removal was verified using ‘vcountPattern’ using the Biostrings package (v2.72.0; Pagès et al. 2024). The remaining sequences were dereplicated, and error rates were estimated using the ‘learnErrors’ function with ‘nbases = 1e8’, leveraging a large dataset to improve error model accuracy. Sample inference was conducted with ‘pool = “pseudo”’, which allows pseudo‐pooling across samples to improve sensitivity for rare variants. Paired‐end reads were merged with a minimum overlap of 10 bases. Chimeric sequences were identified and removed using the ‘removeBimeraDenovo’ function with the ‘consensus’ method to detect chimeras across all samples. The ‘allowOneOff = TRUE’ parameter was used to permit slight mismatches in percent sequences. Additionally, ‘minFoldParentOverAbundance = 2’ was applied to ensure that percent sequences were at least twice as abundant as potential chimeras, providing a conservative yet reliable approach to chimera removal. These steps produced the final unique amplicon sequence variant (ASVs) table. Finally, for taxonomic classification, the ‘assignTaxonomy’ function in DADA2 was used with the SILVA 138.1 database (Mclaren and Callahan 2021), adding species‐level annotation using the ‘addSpecies’ function. All analyses were conducted within v4.4.2 of R Studio (R Core Team 2024).

### 2.6 Data Processing

The ASV tables and taxonomic data generated from the DADA2 pipeline were processed in R Studio to create phyloseq objects using the phyloseq package (v1.48.0; Mcmurdie and Holmes 2013). Representative sequences were added as DNAStringSet objects to the phyloseq object for further integration. A placeholder phylogenetic tree was generated using the ‘rtree’ function in the ape package (v5.8; Paradis and Schliep 2019) and incorporated into the phyloseq objects, ensuring the tree tip labels corresponded to the ASV names. The data were subsequently filtered to retain only bacterial sequences, excluding chloroplast and mitochondrial ASVs. Additionally, samples with zero counts across all ASVs were then removed to prevent empty samples from impacting subsequent analyses. The resulting phyloseq objects were then used for downstream statistical analysis.

Sequencing depths across samples ranged from 391 to 11,565 reads (Table S3). Rarefaction curves (observed ASVs versus sequencing depth) were generated to assess sequencing depth adequacy and comparability across samples (Figure S1). Sequencing depth did not differ significantly among treatment groups (Kruskal–Wallis test, *χ* ^2^ = 1.003, df = 3, *p* = 0.8), supporting balanced comparison across treatments. Because this study aimed to compare bacterial community patterns across treatments and sampling days rather than to exhaustively characterise total soil bacterial diversity, downstream analyses were conducted without rarefaction.

### 2.7 Statistical Analysis

The impact of imidacloprid, thiamethoxam, and clothianidin on bacterial community composition and diversity was assessed by calculating alpha diversity using the Shannon, Simpson (1‐D), Chao1 and ACE indices. These indices were used to measure community richness and evenness across different treatment groups over time. Before conducting the main analysis, Shapiro–Wilk tests were used to assess normality, and Levene's tests were employed to check for homogeneity of variances. While some combinations of Treatment and Day showed normality (*p* > 0.05), others exhibited significant deviations from normality (*p* < 0.05). Despite these deviations, Generalised Linear Models (GLMs) without random effects were fitted using a Gamma distribution with a log link function in the ‘glmmTMB’ package (v1.1.10; Brooks et al. 2017). Day 1 (pre‐treatment) was used as the baseline within each treatment, while the control group served as the baseline across treatments. Model fit was evaluated by examining residuals, checking for overdispersion, and comparing models using AIC (Akaike Information Criterion) and BIC (Bayesian Information Criterion) values. Post hoc comparisons were performed using estimated marginal means in the ‘emmeans’ package (v1.10.6; Lenth 2024) to explore significant differences within treatments over time and between treatments on the same day.

Relative abundance data were aggregated at the phylum level to explore broad taxonomic shifts across treatments. Log‐transformed data were tested for normality using the Shapiro–Wilk test. Phyla with normally distributed data (Shapiro–Wilk *p* ≥ 0.05) were analysed using a two‐way ANOVA to assess the main effects of day and treatment, while those with non‐normal distributions (Shapiro–Wilk *p* < 0.05) were analysed using the non‐parametric Kruskal‐Wallis test. Due to low variability and the presence of many zero values, we focused on the main effects rather than interactions to enhance the usefulness and interpretability of results. This analysis was performed using the packages ‘phyloseq’ (v1.48.0; Mcmurdie and Holmes 2013), ‘dplyr’ (v1.1.4; Wickham et al. 2023), ‘car’ (v3.1.3; Fox and Weisberg 2019), and ‘ggplot2’ (v3.5.1; Wickham 2016).

Beta diversity was assessed using Bray–Curtis dissimilarity calculated at the ASV level computed with the ‘phyloseq’ package (v1.48.0; Mcmurdie and Holmes 2013), and the PERMANOVA test was applied using the ‘adonis2’ function from the ‘vegan’ package (v2.6.6.1; Oksanen et al. 2024) to evaluate the impact of treatment, time, and their interaction on microbial community structure.

To identify key taxa that were differentially abundant between treatment groups, pairwise differential abundance analysis was performed using the Linear Discriminant Analysis Effect Size (LEfSe) method with the function ‘run_lefse’ from the ‘microbiomeMarker’ package (v1.10.0; Cao et al. 2022) to identify taxa significantly enriched between control and treatment groups based on Linear Discriminant Analysis (LDA) scores. Taxa with LDA scores ≥ 3.0 and *p* ≤ 0.05 were considered discriminative biomarkers.

Following the identification of differentially abundant taxa, we investigated how neonicotinoid treatments influenced bacterial interactions and family‐level abundances over time. Spearman's rank correlation was used to explore the relationships between bacterial families and experimental factors such as treatment and sampling day. This analysis was conducted using the ‘microViz’ package (v0.12.5; Barnett et al. 2021).

Alpha diversity indices were visualised using estimated marginal means (EMM) line plots to compare microbial diversity across treatments and sampling days. Relative abundance bar plots were used to present bacterial composition at the phylum level. Principal Coordinates Analysis (PCoA) plots based on Bray‐Curtis distance matrices were used to illustrate shifts in bacterial community structure. LDA score plots from LEfSe analysis were used to visualise differentially abundant taxa between treated and control groups. A correlation heatmap, coupled with a dendrogram, displayed Spearman's rank correlations between bacterial families and experimental factors, providing insights into the relationships among taxa and their responses to neonicotinoid treatments.

## 3 Results

### 3.1 Community Composition and Diversity Patterns

Alpha diversity metrics showed limited overall variation across neonicotinoid treatments and sampling days (Table 1). The generalised linear model showed that significant differences in bacterial diversity occurred across treatments and time; it detected no significant main effects of Treatment or Day, and no significant Treatment × Day interactions for Shannon, Simpson, Chao1, or ACE indices. Notwithstanding the absence of significant global effects, post hoc pairwise comparisons indicated a small number of transient, day‐specific differences relative to the control within individual sampling days (Figures S2–S3). Shannon diversity, a metric reflecting both the richness and evenness of microbial communities (Figure S2a), revealed that thiamethoxam and clothianidin significantly increased microbial diversity compared to the control. Specifically, thiamethoxam resulted in a significant increase in evenness on day 2 (*p* = 0.04), while clothianidin similarly increased diversity on day 3 (*p* = 0.05). In contrast, imidacloprid showed a significant decrease in Shannon diversity compared to both day 1 and the control on day 10 (*p* = 0.01).

**TABLE 1:** Results of Type II Wald chi‐square analysis of variance (ANOVA) from generalised linear models (GLMs) assessing the effects of treatment, sampling day, and their interaction on bacterial alpha diversity indices.

Diversity index	Treatment group			Sampling day			Treatment group × sampling day		
	*χ* ^2^	df	*p*	*χ* ^2^	df	*p*	*χ* ^2^	df	*p*
Shannon	1.5	3	0.6	3.1	5	0.6	15.2	15	0.4
Simpson	3.1		0.3	4.8		0.4	20.9		0.1
Chao1	0.3		0.9	4.9		0.4	18.8		0.2
ACE	0.3		0.9	4.6		0.4	19.0		0.2

The Simpson index, a measure of community dominance (Figure S2b), revealed consistent trends. Thiamethoxam and imidacloprid both significantly increased Simpson's D on Days 2 and 3, respectively (*p* = 0.03), suggesting reduced evenness at early stages. However, by Day 10, Simpson's D significantly decreased under imidacloprid (*p* = 0.01), indicating a subsequent loss of evenness. This suggests that while imidacloprid initially promoted a more even community distribution, dominance by a few taxa became stronger again by Day 10. Clothianidin increased estimated species richness, as indicated by higher Chao1 (Figure S3a) and ACE indices (Figure S3b) on Day 3 compared to the control (*p* = 0.03).

### 3.2 Taxonomic Distribution Shifts

**FIGURE 1:**
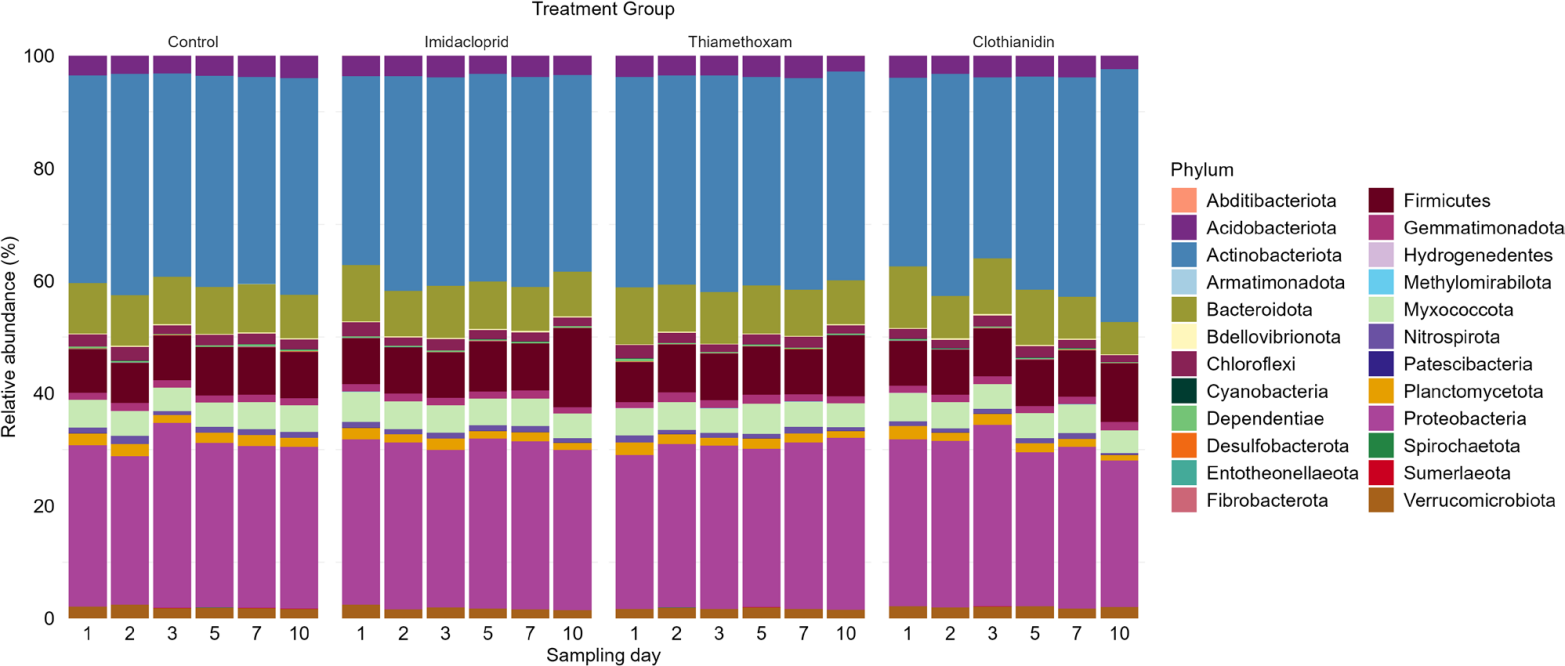
Phylum‐level taxonomic distribution and temporal variation in bacterial relative abundance (%) across different treatments.

The bacterial community in the Red Luvisol soil investigated for this research was comprised of 24 different phyla, of which Actinobacteriota consistently dominated across all treatments (Figure 1). Across all treatments, the five most abundant phyla, with a relative abundance exceeding 5%, were Actinobacteriota (32%–45%), Proteobacteria (27%–33%), Firmicutes (7%–14%), Bacteroidota (6%–11%), and Myxococcota (4%–5%).

The analysis of bacterial community composition at the phylum level revealed stable patterns for dominant phyla and selective changes among minor phyla (Tables S4 and S5). Actinobacteriota and Proteobacteria, the most abundant phyla, showed no significant effects of treatment or sampling day (*p* > 0.05). Firmicutes significantly increased over time (*p* = 0.02), with the highest relative abundance observed under imidacloprid on Day 10, though treatment effects were non‐significant. Bacteroidota significantly decreased over time (*p* = 0.01) without treatment effects.

Among the minor phyla, Methylomirabilota exhibited a significant treatment effect (*p* = 0.001), while Desulfobacterota and Chloroflexi showed low relative abundances with slight, non‐significant decreasing trends over time (*p* > 0.05). Other phyla, Planctomycetota, Abditibacteriota and Verrucomicrobiota, remained stable across treatments and sampling days, with no significant changes detected.

### 3.3 Community Structural Dynamics

Sampling days significantly influenced the structure of bacterial communities (*R* ^2^ = 0.05, *p* = 0.01, Table S6). Moreover, the interaction between treatment and sampling days was also significant (*R* ^2^ = 0.14, *p* = 0.004), indicating that the effect of treatment varied over time. Treatment alone did not significantly affect community structure (*p* > 0.05), suggesting that temporal dynamics and their interaction with treatments were the primary drivers of changes in bacterial community structure.

**FIGURE 2:**
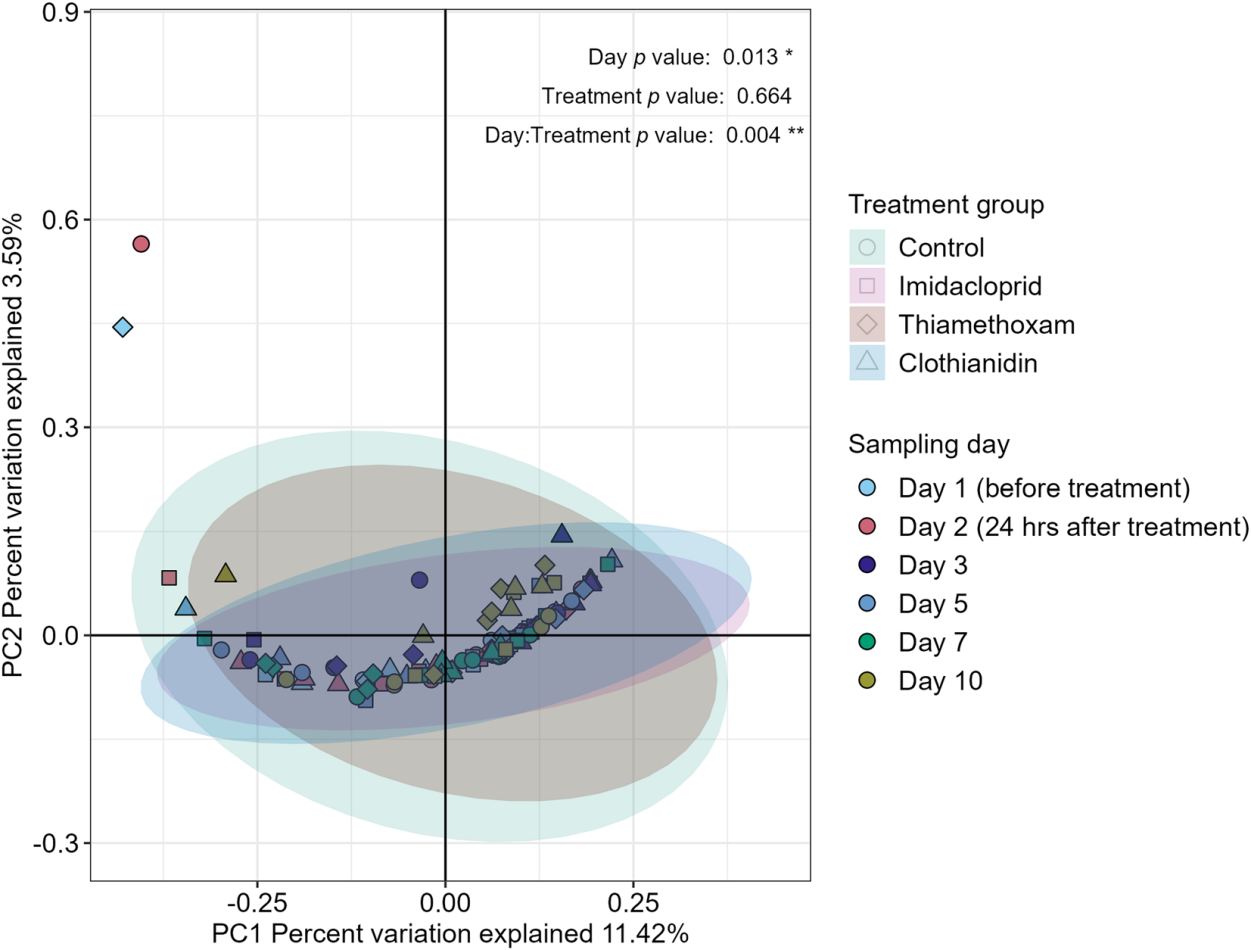
Principal Coordinate Analysis (PCoA) plot of bacterial community structure based on Bray–Curtis dissimilarities at the ASV level. Shapes represented different neonicotinoid treatments, and colours indicated sampling days. Significance levels were set as ‘***’ *p* ≤ 0.001, ‘**’ *p* ≤ 0.01, ‘*’ *p* ≤ 0.05.

The PCoA plots were used to visualise community structural patterns associated with sampling days and their interaction with different neonicotinoids. The first two principal coordinates explained 15.01% of the total variation in the community data, with PC1 accounting for 11.42% and PC2 3.59% of the variation (Figure 2).

Despite the statistical significance, the ellipses for different treatments overlapped substantially, and no distinct clustering patterns were observed. The overlapping ordination space suggests that while temporal changes and their interaction with treatments significantly influenced community structure, the effects were subtle.

### 3.4 Differential Abundance and Key Taxonomic Indicators

**FIGURE 3:**
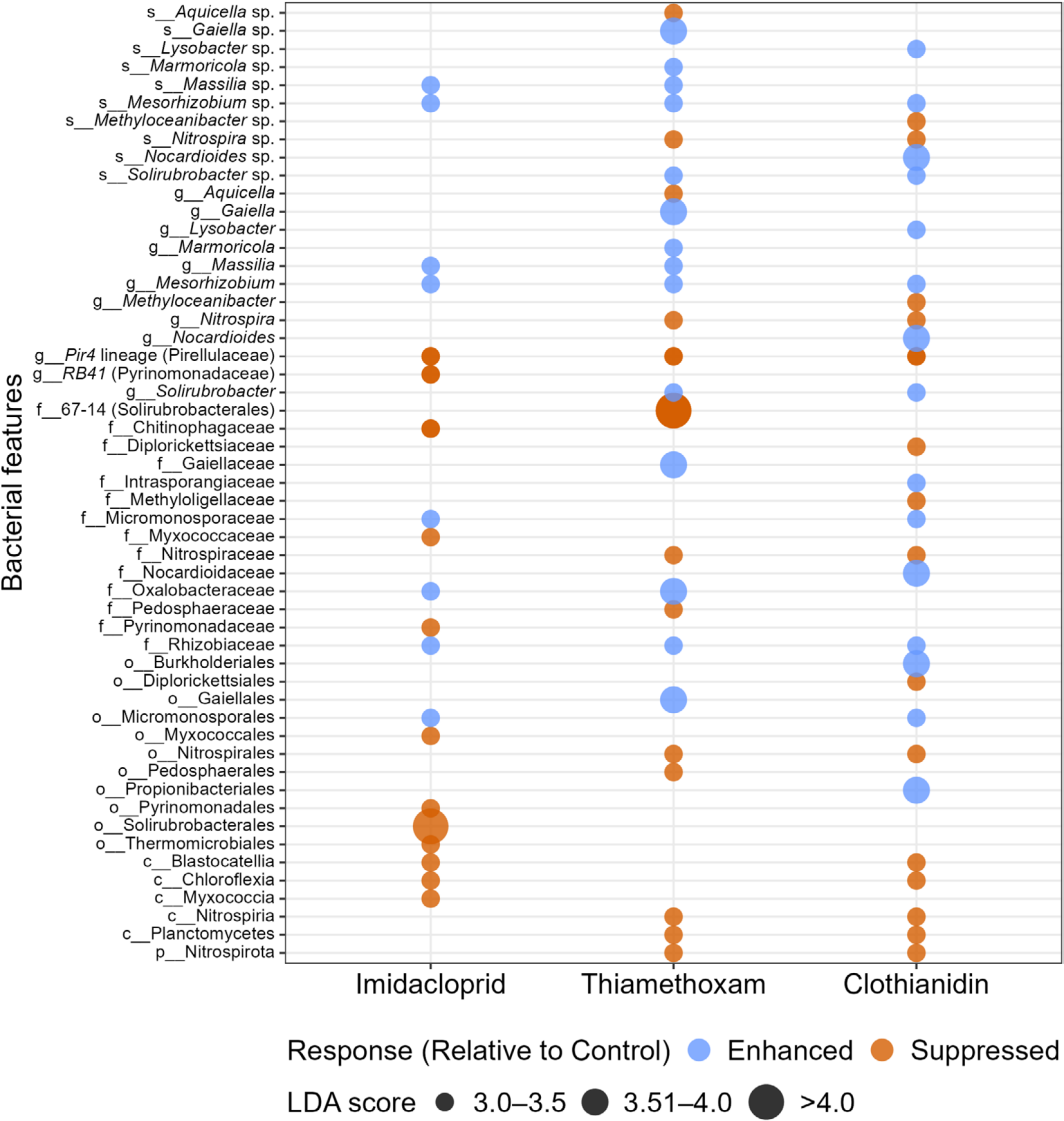
Differential bacterial taxa enriched or suppressed under neonicotinoid treatments relative to the control, identified using LEfSe (LDA ≥ 3.0, *p* ≤ 0.05). Point size reflects the magnitude of the LDA effect size.

The pairwise LEfSe identified significant shifts in the bacterial community structure when comparing control and neonicotinoid‐treated groups. The LDA score plot (Figure 3) illustrated bacterial taxa that were either enhanced or suppressed in relative abundance by the neonicotinoids relative to control across different taxonomic levels. Several taxa showed an enhanced response under imidacloprid, including *Massilia* sp. within Oxalobacteraceae, Micromonosporaceae, and *Mesorhizobium* sp. within the Rhizobiaceae family, with LDA scores ranging from 3.14 to 3.43. Conversely, a broader set of taxa exhibited a suppressed response, including Myxococcaceae, the *Pir4* lineage (Pirellulaceae), *RB41* (Pyrinomonadaceae), Chitinophagaceae, Blastocatellia, Thermomicrobiales, and Solirubrobacterales (LDA = −3.03 to −4.02).

Similarly, thiamethoxam was associated with pronounced bacterial community responses relative to the control. Several taxa exhibited enhanced responses, including members of Gaiellales–Gaiellaceae–*Gaiella* lineage, as well as the families Oxalobacteraceae and Rhizobiaceae, and affiliated genera such as *Solirubrobacter*, *Marmoricola*, *Massilia*, and *Mesorhizobium*, with LDA scores ranging from 3.05 to 3.74. In contrast, thiamethoxam was associated with suppressed responses across multiple lineages, including 67–14 (Solirubrobacterales), Planctomycetes, the Nitrospirota lineage (Nitrospiria–Nitrospirales–Nitrospiraceae–*Nitrospira*), the *Pir4 lineage* (genus‐level taxon within Pirellulaceae), the Pedosphaerales–Pedosphaeraceae lineage, and *Aquicella* (LDA = −3.00 to −4.05).

For clothianidin, enhanced responses were observed for several taxa, including the Propionibacteriales–Nocardioidaceae–Nocardioides lineage, *Solirubrobacter*, Intrasporangiaceae, Rhizobiaceae, Micromonosporales–Micromonosporaceae, *Mesorhizobium* and *Lysobacter* (LDA = 3.01–3.93). Conversely, suppressed responses were detected across multiple bacterial groups, including Chloroflexia, Planctomycetes, *Methyloceanibacter*, the Nitrospirota lineage (Nitrospiria–Nitrospirales–Nitrospiraceae–*Nitrospira*), the Diplorickettsiales–Diplorickettsiaceae lineage, the *Pir4 lineage* (Pirellulaceae), and Blastocatellia (LDA = −3.18 to −3.48).

### 3.5 Correlation Analysis and Taxa Interaction Patterns

**FIGURE 4:**
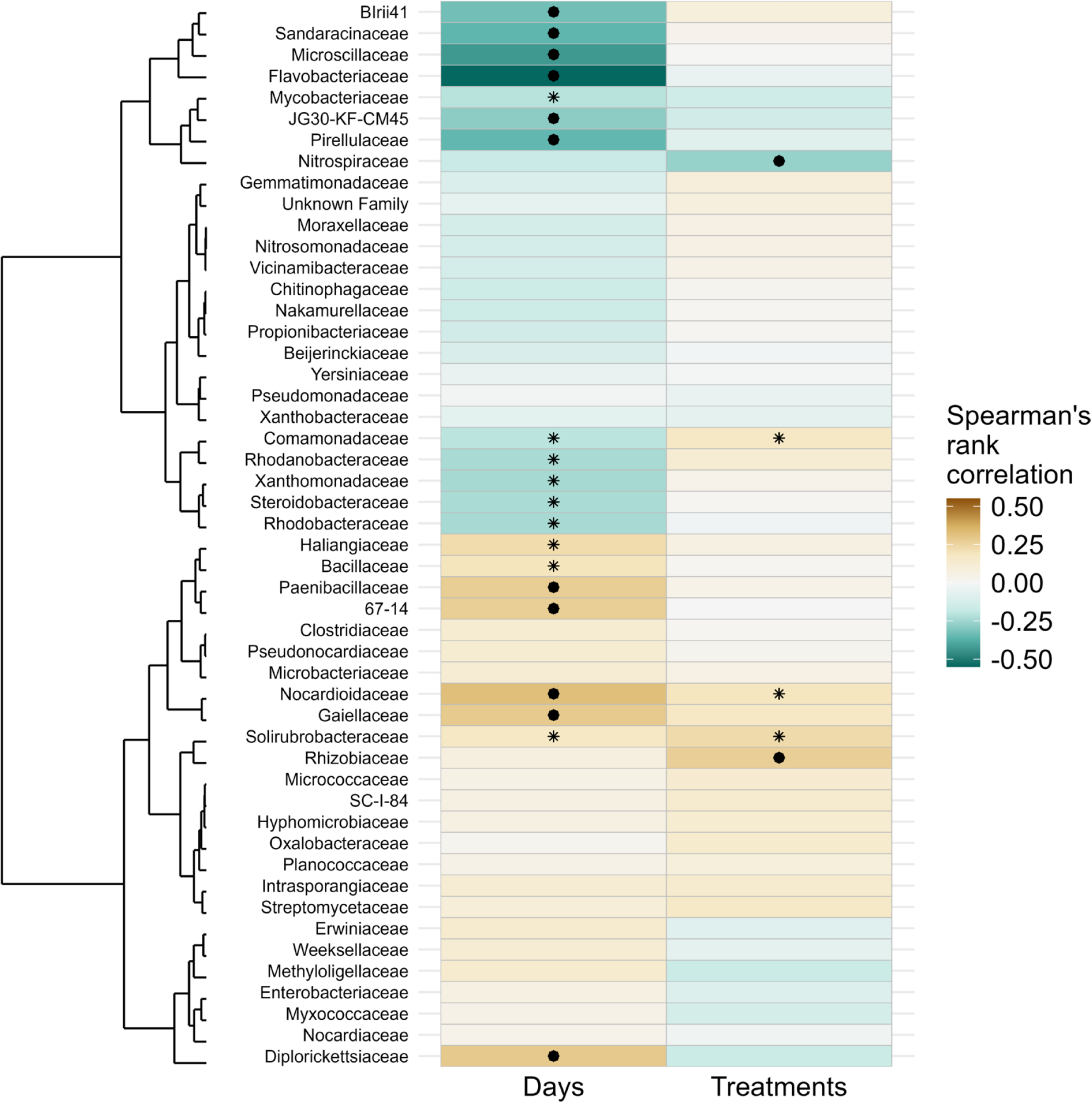
Correlation heatmap and dendrogram showing bacterial family‐level correlations with days and treatments. Significant positive and negative correlations are marked by asterisks (*p* < 0.05, unadjusted), and FDR‐adjusted correlations are highlighted with filled circles (FDR‐corrected *p* < 0.05).

The bacterial correlation heatmap (Figure 4) illustrated how different bacterial families responded to neonicotinoid treatments over time. Several families showed statistically significant correlations with sampling days and treatments; however, correlation strengths were generally weak (|*ρ*| < 0.6), indicating small effect sizes. Representative scatter plots are shown in Figure S4.

67–14 had a positive correlation with days (*ρ* = 0.21, *p* = 0.02), followed by Haliangiaceae (*ρ* = 0.21, *p* = 0.02), Gaiellaceae (*ρ* = 0.24, *p* = 0.01), Weeksellaceae (*ρ* = 0.25, *p* = 0.01), Diplorickettsiaceae (*ρ* = 0.25, *p* = 0.01), Nocardioidaceae (*ρ* = 0.27, *p* = 0.003), Paenibacillaceae (*ρ* = 0.27, *p* = 0.003), and Erwiniaceae (*ρ* = 0.29, *p* = 0.001). Whereas Solirubrobacteraceae (*ρ* = 0.20, *p* = 0.03) and Rhizobiaceae (*ρ* = 0.25, *p* = 0.01) had a positive correlation with treatments. Conversely, a total of 13 microbial families exhibited significant negative correlations with sampling days, indicating that these families decreased in relative abundance over time. Flavobacteriaceae exhibited the strongest negative correlation with sampling days (*ρ* = −0.56, *p* < 0.001). Similarly, Microscillaceae (*ρ* = −0.44, *p* < 0.001) and Sandaracinaceae (*ρ* = −0.41, *p* < 0.001) showed significant negative correlations, indicating a consistent decrease in their abundance over time. Other families such as Pirellulaceae (*ρ* = −0.36, *p* < 0.001), BIrii41 (an uncultured family within Polyangiales; *ρ* = −0.34, *p* < 0.001), and JG30‐KF‐CM45 (an uncultured family within Thermomicrobiales; *ρ* = −0.29, *p* = 0.001) also exhibited negative correlations with sampling days. In addition to the temporal effects, Nitrospiraceae (*ρ* = −0.27, *p* = 0.003) showed a significant negative correlation with treatments, indicating that this family decreased in relative abundance under the applied treatments.

Treatment‐specific analyses revealed that Yersiniaceae (*ρ* = −0.21, *p* = 0.02), 67–14 (*ρ* = −0.20, *p* = 0.03), and Solirubrobacteraceae (*ρ* = −0.19, *p* = 0.03) were significantly negatively correlated with imidacloprid. Mycobacteriaceae (*ρ* = −0.19, *p* = 0.04) was negatively correlated with thiamethoxam treatment, whereas SC‐I‐84 (Burkholderiales) exhibited a positive correlation with clothianidin (*ρ* = 0.21, *p* = 0.02).

## 4 Discussion

### 4.1 Shifts in Diversity Patterns of Bacterial Community Across Neonicotinoid Treatments

The observed shifts in bacterial diversity across different neonicotinoid treatments provide further insights into how these chemicals influence soil microbial communities over time. However, the lack of extensive literature specifically addressing neonicotinoid effects applied as seed treatments on microbial communities makes it difficult to directly compare these findings. This highlights the novelty of our study, as it contributes new insights into the effect of neonicotinoids on soil bacterial communities. By revealing differential impacts across diversity metrics, our research fills a critical gap in understanding the ecological consequences of these widely used insecticides. Similar studies have shown that the response of microbial communities can vary depending on the diversity metric used (Liu et al. 2023), suggesting that richness and evenness are differentially sensitive to different chemical stresses (Herath et al. 2017; Meena et al. 2020).

The initial increase in microbial diversity observed in the Shannon and Simpson indices under thiamethoxam and clothianidin treatments may reflect the early‐stage responses of the soil microbial communities to pesticide exposure. This phenomenon has been observed in other studies, where microbial communities demonstrated an increase in diversity shortly after pesticide exposure, likely as a result of microbial adaptation and the exploitation of newly available ecological niches (Pertile et al. 2021). During this initial phase, certain microorganisms may thrive by utilising the pesticide as a source of energy, leading to a temporary rise in diversity (Gangola et al. 2023, 2021). One possible mechanism behind this early rise in diversity is the activation of resistance mechanisms in microbial populations. Thiamethoxam and clothianidin may activate efflux pumps and increase bacterial cell membrane permeability, promoting horizontal gene transfer and enabling the rapid spread of resistant strains, thereby temporarily boosting microbial diversity (Qiu et al. 2022). However, this increase in diversity is not necessarily sustained. Over time, the pesticide treatments increasingly favour certain resistant strains, causing the gradual decline of less resistant taxa. This is consistent with the concept of ‘selective pressure’, where only those microorganisms capable of resisting the pesticide survive, reducing the overall diversity within the community (Hawkins et al. 2019; Qiu et al. 2022). As a result, we observed a decrease in diversity over time, particularly under the imidacloprid treatment. This decline in diversity, visible in the Simpson index, suggests that over time, the microbial community becomes more dominated by a few pesticide‐resistant taxa, while the rest of the community is suppressed, a pattern often associated with neonicotinoid exposure. This is in line with findings showing that higher doses of imidacloprid significantly reduced bacterial evenness and richness over time, likely due to the displacement of sensitive species by resistant or imidacloprid‐degrading taxa, with both time and dosage playing key roles in shaping the community (Cycoń et al. 2013).

Clothianidin's impact on species richness, as indicated by the ACE and Chao1 indices on day three, suggests that this compound may promote a broader range of bacterial taxa in the early stages of exposure. The temporary increase in richness may reflect an initial proliferation of opportunistic taxa before community structure begins to stabilise. Although direct studies on clothianidin's effect are limited, similar patterns of increased bacterial richness have been observed with other chemicals. For example, a mixture of acetochlor and atrazine applied to surface soil has been shown to significantly increase ACE and Chao1 indices in deeper soil layers (0.2–0.4 m), suggesting that certain chemical compounds can enhance bacterial richness under specific conditions (Chen et al. 2021). Given the short‐term nature of this study, it is possible that longer exposure times may reveal further changes, such as potential recovery or prolonged impacts. Zhang et al. (2019) reported similar patterns with the organochlorine pesticide 1,3‐Dichloropropene, applied as a pre‐plant fumigant, where the initial decrease in bacterial richness was followed by recovery at later stages. These differential findings of imidacloprid, thiamethoxam, and clothianidin on bacterial diversity underscore the complex interactions between neonicotinoids and soil bacterial communities. Significant differences in bacterial diversity indices across treatments reflect the variability in microbial responses, influenced by factors such as environmental conditions, the reactivity of different neonicotinoids, exposure duration, and the specific composition and resilience of microbial taxa. While short‐term increases in diversity were observed, the long‐term effects of pesticide exposure could lead to a reduction in bacterial diversity, potentially compromising soil health. Although shifts in bacterial diversity occurred with treatments and time, these changes were not uniform. This is consistent with the mode of action of neonicotinoids: they are not classified as broad‐spectrum microbial toxins, and their primary toxicity is directed towards insect nervous systems via nicotinic acetylcholine receptors (Jeschke et al. 2011). Nevertheless, neonicotinoids can influence microbial communities indirectly by altering rhizosphere conditions, modifying root exudation patterns, or exerting selective pressure on sensitive taxa (Cycoń and Piotrowska‐Seget 2015). Therefore, a consistent decline in microbial diversity is not necessarily expected. Instead, the observed temporal and compound‐specific shifts may reflect community restructuring, short‐term functional responses, or the emergence of tolerant taxa. These transient changes, even in the absence of clear directional trends, may have important ecological implications, particularly if they affect keystone taxa or microbial functional capacity (Allison and Martiny 2008).

### 4.2 Phylum‐Level Taxonomic Distribution in Response to Neonicotinoids

The bacterial community composition across different neonicotinoid treatments revealed both stable and shifting community dynamics. While the dominant phyla, such as Actinobacteriota and Proteobacteria, exhibited minimal responses to imidacloprid, thiamethoxam, and clothianidin exposure, notable changes were observed among minor phyla over time. These observations suggest that, although some groups of bacteria remain resilient to pesticide stress, others may undergo selective shifts depending on their tolerance to the chemicals and the dynamic soil environment. The stability of Actinobacteriota and Proteobacteria in the presence of neonicotinoids aligns with findings from previous studies, which indicate that these phyla are often dominant in soil and show strong resilience to neonicotinoid as well as other pesticide applications (Li et al. 2018; Galic et al. 2024). This resilience may be attributed to their metabolic diversity and ecological role in soil biogeochemical processes, where they contribute to biodegrade complex organic compounds, including pesticides, which helps maintain their abundance despite exposure to neonicotinoids (Bhende and Dafale 2023; Mawang et al. 2021; Ma et al. 2022; Wang et al. 2023).

Conversely, the rise in Firmicutes under imidacloprid may be attributed to this phylum's spore‐forming ability, which supports survival in chemically stressed environments (Beskrovnaya et al. 2021; Filippidou et al. 2016). Comparable increases in Firmicutes abundance have been noted in soils treated with other neonicotinoids, such as thiamethoxam (Wu et al. 2021) and Paichongding (Cai et al. 2016) as well as with non‐neonicotinoid chemicals like dithiocarbamates (Feld et al. 2015) and ethanol‐free chloroform (Domínguez‐Mendoza et al. 2014). In contrast, Bacteroidota exhibited a significant decrease in relative abundance over time, a trend that suggests susceptibility to neonicotinoid exposure. This aligns with previous studies that reported a decline in Bacteroidota abundance under paichongding stress conditions, indicating that this phylum may be more vulnerable to the effects of neonicotinoids (Cai et al. 2016). Another study reported that imidacloprid changed the abundance of Bacteroidota, with differences occurring between early and later sampling points (Streletskii et al. 2023). Such declines suggest that their metabolic functions could be disrupted by pesticide stress, leading to a decrease in their abundance. Minor phylum Methylomirabilota exhibited significant treatment effects, suggesting that some less‐abundant microbial groups are more sensitive to neonicotinoid exposure. This aligns with previous research, where fumigation treatment of chloropicrin in soils led to a reduction in the abundance of Methylomirabilota over time (Tekeu et al. 2023). While the specific mechanisms remain unclear, it is possible that the decrease in Methylomirabilota in our study could be related to the stress induced by neonicotinoids, thus disrupting microbial community stability.

### 4.3 Community Structure Responses

This study demonstrates that the interaction between sampling day and neonicotinoid treatment significantly influences bacterial community structure in the soil. These findings suggest that microbial responses to neonicotinoids develop progressively over time, rather than as immediate reactions to treatment alone. Such time‐dependent shifts align with previous research, where temporal variation, alongside factors like habitat or host environment, serves as a critical driver of bacterial community composition (Parizadeh et al. 2021). Similarly, studies on chloropicrin and metam sodium reported that sustained exposure leads to community shifts, supporting the role of prolonged chemical presence in altering microbial dynamics (Sennett et al. 2022). This underscores the importance of temporal dynamics in shaping microbial community structure, especially under prolonged chemical stress, as microbial communities in terrestrial ecosystems can exhibit diverse temporal changes, from immediate responses to seasonal variations (Schmidt et al. 2007; Bardgett et al. 2005).

The PCoA analysis showed that temporal and treatment interactions led to subtle structural changes in bacterial communities, without distinct clustering patterns. This lack of separation may reflect the functional redundancy often seen in microbial communities, where diverse taxa can fulfil similar roles, stabilising function despite taxonomic shifts (Allison and Martiny 2008). Additionally, the complexity of soil microhabitats likely introduces spatial variability, potentially masking distinct clustering in ordination space (Fierer and Jackson 2006). Such subtle responses align with studies indicating that microbial adaptation to stressors, including chemical exposure, tends to accumulate over time rather than producing immediate changes (Shade et al. 2012). Furthermore, the partial variance explained by PCoA suggests that other environmental factors, not captured here, might also influence community dynamics (Ramette 2007). These findings highlight that while the interaction of treatment with three different neonicotinoids and time significantly influences bacterial communities, the resulting shifts are complex and gradual. This suggests that each neonicotinoid may contribute uniquely to changes in soil microbial dynamics, with potential implications for soil ecosystem stability under prolonged chemical exposure.

### 4.4 Taxonomic Indicators of Bacterial Community Response to Neonicotinoids

The differential abundance analysis highlights specific bacterial taxa that respond uniquely to each neonicotinoid treatment, indicating selective effects on bacterial community composition with implications for multiple biogeochemical cycles. Taxa that are important within key biogeochemical cycles benefited from soil exposure to neonicotinoids. All three neonicotinoids led to the enhancement of specific bacterial taxa. *Mesorhizobium*, a genus known for nitrogen fixation (Laranjo et al. 2014; Colombi et al. 2023), was enhanced under all treatments, indicating that neonicotinoids may stimulate nitrogen cycling, which is beneficial for soil fertility and plant growth. Additionally, *Mesorhizobium*'s production of 1‐aminocyclopropane‐1‐carboxylic acid deaminase can help alleviate plant stress by limiting ethylene formation, a hormone that is typically produced in response to stressors such as drought, salinity, and heavy metals (Wanjofu et al. 2022). This enzymatic activity may support plant growth under various environmental stress conditions. Imidacloprid also enriched the Micromonosporaceae family, which is involved in the breakdown of complex organic compounds such as cellulose, chitin, lignin and pectin (Trujillo et al. 2014). These bacteria are also stress‐tolerant, capable of nitrogen fixation and decomposition of carbon compounds, all of which are important for maintaining soil health (Skidmore et al. 2022). Thiamethoxam also enhanced *Massilia* sp., *Gaiella* sp. and *Solirubrobacter* sp., whereas clothianidin enhanced the latter one. The bacterial genus *Massilia* exhibits significant ecological importance through its adaptability across diverse ecosystems, from extreme environments like deserts and glaciers to polluted soils and airborne communities, where it aids in pollutant bioremediation, enhances plant resilience in phytoremediation, and serves as a potential bioindicator of environmental stress (Amirhosseini et al. 2025). *Gaiella* sp. promote the biodegradation of organic pollutants by using intermediate metabolites and is involved in phosphate absorption and organic matter decomposition (Gu et al. 2023; Wei et al. 2024). Similarly, *Solirubrobacter* sp. plays a crucial role in soil organic matter assimilation and biogeochemical cycling and contributes significantly to soil fertility and nutrient availability (Jiang et al. 2023; Jara‐Servin et al. 2024). Clothianidin also enriched *Lysobacter*, a predatory Gammaproteobacterium that employs T4SS‐mediated contact‐dependent killing of gram‐negative soil bacteria (Shen et al. 2021). This enrichment reveals pesticide‐specific selection for microbial predators possessing lytic enzymes and antibiotics that suppress soilborne pathogens while maintaining nutrient cycling, demonstrating functional resilience under neonicotinoid stress.

While some key taxa benefited by soil exposure to neonicotinoids, the suppression of other bacterial taxa is concerning as they too play critical roles in soil health and ecosystem functions. Imidacloprid and clothianidin suppressed Chloroflexia, which is involved in degradation of complex carbohydrates through diverse CAZymes and contributes to carbon cycling (Freches and Fradinho 2024; Zheng et al. 2021). The reduction of Chloroflexia could impair plant‐derived carbohydrate decomposition and slow down carbon cycling in soil (Dobrzyński et al. 2026). The suppression of *Nitrospira* sp. by both thiamethoxam and clothianidin is particularly troubling because these taxa are involved in key nitrogen processes. The suppression of *Nitrospira* sp., responsible for nitrification, further points to potential disruptions in the nitrogen cycle by impairing nitrite oxidation to nitrate, potentially leading to reduced nutrient mineralisation for plants (Takahashi et al. 2020; Meng et al. 2023). Clothianidin also suppressed *Methyloceanibacter* sp., a flexible methylotroph that scavenges C_1_ compounds, participates in methane‐linked food webs via cross‐feeding and couples C_1_ oxidation to nitrogen transformations, especially in moist, redox‐dynamic soil environments (Howland et al. 2025; Rasmussen et al. 2024; Vekeman et al. 2016). This could elevate methane emissions, hinder N‐cycling, and disrupt microbiome stability, underscoring pesticide risks to soil C/N homeostasis (Spokas et al. 2007; Itoh et al. 2014).

### 4.5 Correlation and Taxa Interaction Patterns

The correlation analysis reveals distinct short‐term responses of bacterial families to neonicotinoid treatments, indicating that neonicotinoids influence microbial dynamics in a treatment‐specific and time‐sensitive manner. The positive correlations of 67–14 (*Solirubrobacterales*), *Haliangiaceae, Gaiellaceae, and Weeksellaceae* with sampling days may indicate an initial resilience or adaptation, allowing these families to maintain or slightly increase in abundance under early neonicotinoid exposure. This aligns with findings from other studies showing that certain bacterial taxa can exhibit resilience to pesticide stress, potentially due to metabolic flexibility or adaptive mechanisms (Shahid et al. 2023; Jiao et al. 2019). In contrast, Flavobacteriaceae and Microscillaceae exhibited negative correlations with days, suggesting short‐term sensitivity to neonicotinoid stress or competitive interactions within the microbial community. This variability in bacterial resilience highlights the influence of different chemical treatments, with certain microbial groups being more sensitive to specific pesticides, which can affect their roles in maintaining ecosystem stability (Meena et al. 2020).

Beyond the general trends that were observed, each treatment had unique effects on specific bacterial families. The significant negative correlation of Yersiniaceae with imidacloprid suggests that this family may be sensitive to neonicotinoid exposure. Despite its pathogenic significance (Seabaugh and Anderson 2024), there is a lack of evidence regarding the ecological importance of Yersiniaceae in soil ecosystems. Similarly, the negative correlation of 67–14 with imidacloprid indicates its susceptibility to neonicotinoid exposure. Given its positive correlation with microbial biomass carbon and specific surface area, the suppression of 67–14 may reflect disruptions in key soil processes, such as carbon cycling and the maintenance of soil structure, which are essential for soil health and microbial community stability (Company et al. 2022). The negative correlation of Solirubrobacteraceae with imidacloprid aligns with previous research where this family was significantly depleted by chloropicrin applied as a soil fumigant (Zhan et al. 2021). As a family associated with the rhizosphere, Solirubrobacteraceae is likely involved in plant nutrition and soil health (Jiang et al. 2023). While its specific functional roles are unclear, it is known to possess broad ecological capabilities, thriving in both aquatic and terrestrial environments, including disturbed soils (Evdokimova et al. 2024). Similarly, Mycobacteriaceae, recognised for its role in the degradation of hydrocarbons and pollutants (Conte et al. 2021; Alidoosti et al. 2024), was negatively correlated with thiamethoxam in our study, suggesting its sensitivity to neonicotinoid exposure. This finding supports previous studies that demonstrated that a non‐ionic surfactant hindered the proliferation of Mycobacteriaceae, affecting its ability to degrade polycyclic aromatic hydrocarbons (Lladó et al. 2015). Thus, Mycobacteriaceae may also be sensitive to neonicotinoids, whose presence in soil may reduce their efficacy in biodegradation processes. In contrast, clothianidin was positively correlated with SC‐I‐84 (Burkholderiales). This aligns with findings from previous studies, which suggest that pollutants such as Ni, Cu, Pb, and organic contaminants favour the growth of SC‐I‐84 (Wang et al. 2024).

We recommend future research focus on long‐term studies to assess cumulative neonicotinoid effects on microbial community composition, particularly in relation to soil ecosystem functions. Comparative analyses of individual neonicotinoids could further clarify how each compound differentially impacts soil microbial composition, diversity, and function. Additionally, incorporating environmental parameters such as soil pH, moisture, organic matter, and texture could provide insights into the interactions between neonicotinoids and natural soil conditions, enhancing the understanding of microbial stability and resilience under varying field conditions. Mechanistic studies examining metabolic pathways and adaptation responses in neonicotinoid‐affected taxa are also essential for advancing knowledge of how prolonged chemical exposure influences soil microbial networks, functional resilience, and specific ecological roles. A more functional perspective could reveal the broader implications of microbial shifts, shedding light on how these communities sustain or alter critical soil processes over extended exposure periods.

## 5 Conclusion

Our results showed that neonicotinoid seed treatments altered microbial diversity, taxonomic composition, and community structure during short‐term exposure. Each neonicotinoid induced unique shifts in bacterial diversity and taxa interactions, with evidence of both resilience and sensitivity among bacterial taxa. Initial increases in diversity under thiamethoxam and clothianidin were followed by declines, particularly under imidacloprid, suggesting progressive selective pressure during continued exposure. Although dominant phyla remained relatively stable, less abundant taxa showed sensitivity, and community changes over time suggest that continued exposure favours stress‐tolerant populations. The enrichment of genera *Mesorhizobium*, *Massilia*, *Gaiella, Solirubrobacter*, and *Lysobacter* suggests adaptive or stress‐tolerant capacities within certain taxa, whereas the suppression of Chloroflexia, *Nitrospira*, and *Methyloceanibacter* points to potential vulnerabilities in carbon and nitrogen cycling pathways. Given the limited body of literature on neonicotinoid seed treatments, this research provides insights into early soil microbial responses. Further long‐term and functional investigations are needed to elucidate metabolic pathways, functional adaptations, and resilience mechanisms under repeated and cumulative neonicotinoid exposure across soils with differing chemical and physical properties.

## Author Contributions

**Sharmin Akter:** conceptualisation, methodology, investigation, laboratory analysis, data analysis, data curation, visualisation, writing – original draft. **Julia Jasonsmith:** supervision, method validation, writing – review and editing. **Nilantha R. Hulugalle:** supervision, method validation, writing – review and editing. **Craig L. Strong:** supervision, method validation, writing – review and editing.

## Funding

This work was supported by Australian National University, ANU Research Scholarship (International), ANU HDR Fee Remission Merit Scholarship.

## Conflicts of Interest

The authors declare no conflicts of interest.

## Supporting information


**Table S1:** Summary of physicochemical properties of the soil samples analysed in this study.
**Table S2:**. Primer sequences used for amplification of the 16S rRNA gene (V1–V3 region).
**Table S3:**. Sequencing depths by treatments.
**Table S4:**. Bacterial relative abundance (%) at the phylum level across different treatments over time.
**Table S5:**. Statistical test of different bacterial phyla across different treatments and time, with effect direction assessed using median and negative significance highlighted by the red circle and positive significance highlighted by the green circle.
**Table S6:**. PERMANOVA results showing the influence of different neonicotinoid treatments, sampling day, and their interaction on soil bacterial community structure measured by Bray–Curtis dissimilarity matrix.
**Figure S1:**. Rarefaction curves showing sequencing depth for each sample.
**Figure S2:**. Alpha diversity indices for bacteria, measured by (a) Shannon index, (b) Simpson index. Points represent estimated marginal means (EMMs) ± 95% confidence intervals for each treatment group (Control, Imidacloprid, Thiamethoxam, and Clothianidin). Asterisks indicate significant differences among treatments within each day based on Generalised Linear Model analysis (‘**’ ≤ 0.01, ‘*’ ≤ 0.05).
**Figure S3:**. Alpha diversity indices for bacteria, evaluated through (a) Chao1 index, (b) ACE index. Points represent estimated marginal means (EMMs) ±95% confidence intervals for each treatment group (Control, Imidacloprid, Thiamethoxam and Clothianidin). Asterisks indicate significant differences among treatments within each day based on Generalised Linear Model analysis (‘*’ ≤ 0.05).
**Figure S4:**. Scatter plot showing CLR‐transformed relative abundance of bacterial families that showed significant Spearman correlations (*p* ≤ 0.05) with sampling day and/or neonicotinoid treatments.

## Data Availability

The raw sequencing data produced in this study have been deposited in the NCBI Sequence Read Archive under accession number PRJNA1251256. Other datasets relevant to the findings of this article are provided within the main text and the accompanying Supporting Information files.

## References

[emi470339-bib-0001] Akter, S. , N. R. Hulugalle , J. Jasonsmith , and C. L. Strong . 2023. “Changes in Soil Microbial Communities After Exposure to Neonicotinoids: A Systematic Review.” Environmental Microbiology Reports 15, no. 6: 431–444. 10.1111/1758-2229.13193. 37574328 PMC10667664

[emi470339-bib-0002] Alidoosti, F. , M. Giyahchi , S. Moien , and H. Moghimi . 2024. “Unlocking the Potential of Soil Microbial Communities for Bioremediation of Emerging Organic Contaminants: Omics‐Based Approaches.” Microbial Cell Factories 23, no. 1: 210. 10.1186/s12934-024-02485-z. 39054471 PMC11271216

[emi470339-bib-0003] Allison, S. D. , and J. B. H. Martiny . 2008. “Resistance, Resilience, and Redundancy in Microbial Communities.” Proceedings of the National Academy of Sciences 105: 11512–11519. 10.1073/pnas.0801925105. PMC2556421 18695234

[emi470339-bib-0004] Alori, E. T. , B. R. Glick , and O. O. Babalola . 2017. “Microbial Phosphorus Solubilization and Its Potential for Use in Sustainable Agriculture.” Frontiers in Microbiology 8: 971. 10.3389/fmicb.2017.00971. 28626450 PMC5454063

[emi470339-bib-0005] Amirhosseini, K. , M. Alizadeh , and H. Azarbad . 2025. “Harnessing the Ecological and Genomic Adaptability of the Bacterial Genus *Massilia* for Environmental and Industrial Applications.” Microbial Biotechnology 18, no. 5: e70156. 10.1111/1751-7915.70156. 40325956 PMC12053321

[emi470339-bib-0006] Bakker, L. , W. Van Der Werf , P. Tittonell , K. A. G. Wyckhuys , and F. J. J. A. Bianchi . 2020. “Neonicotinoids in Global Agriculture: Evidence for a New Pesticide Treadmill?” Ecology and Society 25, no. 3: 26. 10.5751/ES-11814-250326.

[emi470339-bib-0007] Bardgett, R. D. , W. D. Bowman , R. Kaufmann , and S. K. Schmidt . 2005. “A Temporal Approach to Linking Aboveground and Belowground Ecology.” Trends in Ecology & Evolution 20, no. 11: 634–641. 10.1016/j.tree.2005.08.005. 16701447

[emi470339-bib-0008] Barnett, D. J. M. , I. C. W. Arts , and J. Penders . 2021. “microViz: An R Package for Microbiome Data Visualization and Statistics.” Journal of Open Source Software 6, no. 63: 3201. 10.21105/joss.03201.

[emi470339-bib-0009] Basu, S. , G. Kumar , S. Chhabra , and R. Prasad . 2021. “Role of Soil Microbes in Biogeochemical Cycle for Enhancing Soil Fertility.” In New and Future Developments in Microbial Biotechnology and Bioengineering, edited by J. P. Verma , C. A. Macdonald , V. K. Gupta , and A. R. Podile , 149–157. Elsevier. 10.1016/B978-0-444-64325-4.00013-4.

[emi470339-bib-0010] Bayer Cropscience . 2023. “Poncho 600 FS Seed Treatment Insecticide Label.” https://agriculture.basf.ca/east/en/products/solutions/poncho‐600‐fs.html.

[emi470339-bib-0011] Bayer Cropscience Australia . 2023. “Gaucho 600 Red Flowable Seed Treatment Insecticide Label.” https://www.crop.bayer.com.au/‐/media/bcs‐inter/ws_australia/use‐our‐products/product‐import‐files/168/gaucho‐600‐product‐label.pdf.

[emi470339-bib-0012] Beskrovnaya, P. , D. Fakih , I. Morneau , et al. 2021. “No Endospore Formation Confirmed in Members of the Phylum Proteobacteria.” Applied and Environmental Microbiology 87, no. 5: e02312‐20. 10.1128/AEM.02312-20. 33355101 PMC8090866

[emi470339-bib-0013] Bhende, R. S. , and N. A. Dafale . 2023. “Insights Into the Ubiquity, Persistence and Microbial Intervention of Imidacloprid.” Archives of Microbiology 205, no. 5: 215. 10.1007/s00203-023-03516-w. 37129684

[emi470339-bib-0014] Bonmatin, J.‐M. , C. Giorio , V. Girolami , et al. 2015. “Environmental Fate and Exposure; Neonicotinoids and Fipronil.” Environmental Science and Pollution Research 22, no. 1: 35–67. 10.1007/s11356-014-3332-7. 25096486 PMC4284396

[emi470339-bib-0015] Bonmatin, J.‐M. , E. a. D. Mitchell , G. Glauser , et al. 2021. “Residues of Neonicotinoids in Soil, Water and People's Hair: A Case Study From Three Agricultural Regions of The Philippines.” Science of the Total Environment 757: 143822. 10.1016/j.scitotenv.2020.143822. 33246718

[emi470339-bib-0016] Briceño, G. , M. C. Diez , G. Palma , et al. 2024. “Neonicotinoid Effects on Soil Microorganisms: Responses and Mitigation Strategies.” Sustainability 16, no. 9: 3769. 10.3390/su16093769.

[emi470339-bib-0017] Brooks, M. E. , K. Kristensen , K. J. V. Benthem , et al. 2017. “glmmTMB Balances Speed and Flexibility Among Packages for Zero‐Inflated Generalized Linear Mixed Modeling.” R Journal 9, no. 2: 378–400. 10.32614/RJ-2017-066.

[emi470339-bib-0018] Buszewski, B. , M. Bukowska , M. Ligor , and I. Staneczko‐Baranowska . 2019. “A Holistic Study of Neonicotinoids Neuroactive Insecticides—Properties, Applications, Occurrence, and Analysis.” Environmental Science and Pollution Research 26, no. 34: 34723–34740. 10.1007/s11356-019-06114-w. 31520389 PMC6900273

[emi470339-bib-0019] Cai, Z. , J. Ma , J. Wang , J. Cai , G. Yang , and X. Zhao . 2016. “Impact of the Novel Neonicotinoid Insecticide Paichongding on Bacterial Communities in Yellow Loam and Huangshi Soils.” Environmental Science and Pollution Research 23, no. 6: 5134–5142. 10.1007/s11356-015-5733-7. 26552792

[emi470339-bib-0020] Callahan, B. J. , P. J. Mcmurdie , M. J. Rosen , A. W. Han , A. J. A. Johnson , and S. P. Holmes . 2016. “DADA2: High‐Resolution Sample Inference From Illumina Amplicon Data.” Nature Methods 13, no. 7: 581–583. 10.1038/nmeth.3869. 27214047 PMC4927377

[emi470339-bib-0021] Cao, Y. , Q. Dong , D. Wang , P. Zhang , Y. Liu , and C. Niu . 2022. “microbiomeMarker: An R/Bioconductor Package for Microbiome Marker Identification and Visualization.” Bioinformatics 38, no. 16: 4027–4029. 10.1093/bioinformatics/btac438. 35771644

[emi470339-bib-0022] Castillo‐Díaz, J. M. , F. Martin‐Laurent , J. Beguet , R. Nogales , and E. Romero . 2017. “Fate and Effect of Imidacloprid on Vermicompost‐Amended Soils Under Dissimilar Conditions: Risk for Soil Functions, Structure, and Bacterial Abundance.” Science of the Total Environment 579: 1111–1119. 10.1016/j.scitotenv.2016.11.082. 27914643

[emi470339-bib-0023] Chen, J. , W. Yang , J. Li , et al. 2021. “Effects of Herbicides on the Microbial Community and Urease Activity in the Rhizosphere Soil of Maize at Maturity Stage.” Journal of Sensors 2021, no. 1: 6649498. 10.1155/2021/6649498.

[emi470339-bib-0024] Colombi, E. , Y. Hill , R. Lines , et al. 2023. “Population Genomics of Australian Indigenous *Mesorhizobium* Reveals Diverse Nonsymbiotic Genospecies Capable of Nitrogen‐Fixing Symbioses Following Horizontal Gene Transfer.” Microbial Genomics 9, no. 1: 000918. 10.1099/mgen.0.000918. PMC9973854 36748564

[emi470339-bib-0025] Company, J. , N. Valiente , J. Fortesa , et al. 2022. “Secondary Succession and Parent Material Drive Soil Bacterial Community Composition in Terraced Abandoned Olive Groves From a Mediterranean Hyper‐Humid Mountainous Area.” Agriculture, Ecosystems & Environment 332: 107932. 10.1016/j.agee.2022.107932.

[emi470339-bib-0026] Conte, A. , S. Chiaberge , F. Pedron , et al. 2021. “Dealing With Complex Contamination: A Novel Approach With a Combined Bio‐Phytoremediation Strategy and Effective Analytical Techniques.” Journal of Environmental Management 288: 112381. 10.1016/j.jenvman.2021.112381. 33823438

[emi470339-bib-0027] Cycoń, M. , A. Markowicz , S. Borymski , M. Wójcik , and Z. Piotrowska‐Seget . 2013. “Imidacloprid Induces Changes in the Structure, Genetic Diversity and Catabolic Activity of Soil Microbial Communities.” Journal of Environmental Management 131: 55–65. 10.1016/j.jenvman.2013.09.041. 24140487

[emi470339-bib-0028] Cycoń, M. , and Z. Piotrowska‐Seget . 2015. “Biochemical and Microbial Soil Functioning After Application of the Insecticide Imidacloprid.” Journal of Environmental Sciences 27: 147–158. 10.1016/j.jes.2014.05.034. 25597673

[emi470339-bib-0029] Dini‐Andreote, F. , and J. D. Van Elsas . 2019. “The Soil Microbiome‐An Overview.” In Modern Soil Microbiology, edited by J. D. Van Elsas , J. T. Trevors , A. S. Rosado , and P. Nannipieri , 3rd ed., 37–48. CRC Press. 10.1201/9780429059186.

[emi470339-bib-0030] Dobrzyński, J. , M. Gradowski , A. Radkowski , and H. Bujak . 2026. “Chloroflexota in Agricultural Soils: Current Knowledge and Future Research Directions.” Frontiers in Microbiology 17: 1705889. 10.3389/fmicb.2026.1705889. 41695957 PMC12901349

[emi470339-bib-0031] Domínguez‐Mendoza, C. A. , J. M. Bello‐López , Y. E. Navarro‐Noya , et al. 2014. “Bacterial Community Structure in Fumigated Soil.” Soil Biology and Biochemistry 73: 122–129. 10.1016/j.soilbio.2014.02.012.

[emi470339-bib-0032] Evdokimova, E. , E. Ivanova , G. Gladkov , et al. 2024. “Structural Shifts in the Soil Prokaryotic Communities Marking the Podzol‐Forming Process on Sand Dumps.” Soil Systems 8, no. 1: 9. 10.3390/soilsystems8010009.

[emi470339-bib-0033] Feld, L. , M. H. Hjelmsø , M. S. Nielsen , et al. 2015. “Pesticide Side Effects in an Agricultural Soil Ecosystem as Measured by amoA Expression Quantification and Bacterial Diversity Changes.” PLoS One 10, no. 5: e0126080. 10.1371/journal.pone.0126080. 25938467 PMC4418756

[emi470339-bib-0034] Fierer, N. 2017. “Embracing the Unknown: Disentangling the Complexities of the Soil Microbiome.” Nature Reviews Microbiology 15, no. 10: 579–590. 10.1038/nrmicro.2017.87. 28824177

[emi470339-bib-0035] Fierer, N. , and R. B. Jackson . 2006. “The Diversity and Biogeography of Soil Bacterial Communities.” Proceedings of the National Academy of Sciences 103, no. 3: 626–631. 10.1073/pnas.0507535103. PMC1334650 16407148

[emi470339-bib-0036] Filippidou, S. , T. Wunderlin , T. Junier , et al. 2016. “A Combination of Extreme Environmental Conditions Favor the Prevalence of Endospore‐Forming Firmicutes.” Frontiers in Microbiology 7: 1707. 10.3389/fmicb.2016.01707. 27857706 PMC5094177

[emi470339-bib-0037] Fox, J. , and S. Weisberg . 2019. An R Companion to Applied Regression. SAGE Publications.

[emi470339-bib-0038] Freches, A. , and J. C. Fradinho . 2024. “The Biotechnological Potential of the Chloroflexota Phylum.” Applied and Environmental Microbiology 90, no. 6: e01756‐23. 10.1128/aem.01756-23. 38709098 PMC11218635

[emi470339-bib-0039] Galic, I. , C. Bez , I. Bertani , V. Venturi , and N. Stankovic . 2024. “Herbicide‐Treated Soil as a Reservoir of Beneficial Bacteria: Microbiome Analysis and PGP Bioinoculants in Maize.” Environmental Microbiomes 19, no. 1: 107. 10.1186/s40793-024-00654-6. PMC11657599 39695885

[emi470339-bib-0040] Gangola, S. , S. Joshi , G. Bhandari , et al. 2023. “Exploring Microbial Diversity Responses in Agricultural Fields: A Comparative Analysis Under Pesticide Stress and Non‐Stress Conditions.” Frontiers in Microbiology 14: 1271129. 10.3389/fmicb.2023.1271129. 37928679 PMC10623313

[emi470339-bib-0041] Gangola, S. , S. Joshi , S. Kumar , B. Sharma , and A. Sharma . 2021. “Differential Proteomic Analysis Under Pesticides Stress and Normal Conditions in *Bacillus cereus* 2D.” PLoS One 16, no. 8: e0253106. 10.1371/journal.pone.0253106. 34388169 PMC8362991

[emi470339-bib-0042] Gu, H. , J. Yan , Y. Liu , et al. 2023. “Autochthonous Bioaugmentation Accelerates Phenanthrene Degradation in Acclimated Soil.” Environmental Research 224: 115543. 10.1016/j.envres.2023.115543. 36822540

[emi470339-bib-0043] Gupta, A. , U. B. Singh , P. K. Sahu , et al. 2022. “Linking Soil Microbial Diversity to Modern Agriculture Practices: A Review.” International Journal of Environmental Research and Public Health 19, no. 5: 3141. 10.3390/ijerph19053141. 35270832 PMC8910389

[emi470339-bib-0044] Hawkins, N. J. , C. Bass , A. Dixon , and P. Neve . 2019. “The Evolutionary Origins of Pesticide Resistance.” Biological Reviews 94, no. 1: 135–155. 10.1111/brv.12440. 29971903 PMC6378405

[emi470339-bib-0045] Herath, L. I. , P. Moldrup , L. W. De Jonge , et al. 2017. “Clay‐To‐Carbon Ratio Controls the Effect of Herbicide Application on Soil Bacterial Richness and Diversity in a Loamy Field.” Water, Air, & Soil Pollution 228, no. 1: 3. 10.1007/s11270-016-3175-6.

[emi470339-bib-0046] Hou, D. , N. S. Bolan , D. C. W. Tsang , M. B. Kirkham , and D. O'connor . 2020. “Sustainable Soil Use and Management: An Interdisciplinary and Systematic Approach.” Science of the Total Environment 729: 138961. 10.1016/j.scitotenv.2020.138961. 32353725 PMC7182530

[emi470339-bib-0047] Howland, K. E. , H. J. Nygaard , A. D. Steen , K. M. Halanych , A. R. Mahon , and D. R. Learman . 2025. “Potential for Microbial Denitrification Coupled With Methanol Oxidation Found in Abundant MAGs in Antarctic Peninsula Sediments.” FEMS Microbiology Letters 372: fnaf050. 10.1093/femsle/fnaf050. 40397438

[emi470339-bib-0048] Itoh, H. , R. Navarro , K. Takeshita , et al. 2014. “Bacterial Population Succession and Adaptation Affected by Insecticide Application and Soil Spraying History.” Frontiers in Microbiology 5: 457. 10.3389/fmicb.2014.00457. 25221549 PMC4148734

[emi470339-bib-0049] Iuss Working Group Wrb . 2015. World Reference Base for Soil Resources 2014, Update 2015 International Soil Classification System for Naming Soils and Creating Legends for Soil Maps. FAO.

[emi470339-bib-0050] Jara‐Servin, A. , G. Mejia , M. F. Romero , M. Peimbert , and L. D. Alcaraz . 2024. “Unravelling the Genomic and Environmental Diversity of the Ubiquitous Solirubrobacter.” Environmental Microbiology 26, no. 8: e16685. 10.1111/1462-2920.16685. 39147372

[emi470339-bib-0051] Jeschke, P. , and R. Nauen . 2005. “Neonicotinoid Insecticides.” In Comprehensive Molecular Insect Science, edited by L. I. Gilbert , 53–105. Elsevier. 10.1016/B0-44-451924-6/00069-7.

[emi470339-bib-0052] Jeschke, P. , R. Nauen , M. Schindler , and A. Elbert . 2011. “Overview of the Status and Global Strategy for Neonicotinoids.” Journal of Agricultural and Food Chemistry 59, no. 7: 2897–2908. 10.1021/jf101303g. 20565065

[emi470339-bib-0053] Jiang, Z.‐M. , T. Mou , Y. Sun , J. Su , L.‐Y. Yu , and Y.‐Q. Zhang . 2023. “Environmental Distribution and Genomic Characteristics of Solirubrobacter, With Proposal of Two Novel Species.” Frontiers in Microbiology 14: 1267771. 10.3389/fmicb.2023.1267771. 38107860 PMC10722151

[emi470339-bib-0054] Jiao, S. , W. Chen , and G. Wei . 2019. “Resilience and Assemblage of Soil Microbiome in Response to Chemical Contamination Combined With Plant Growth.” Applied and Environmental Microbiology 85, no. 6: e02523‐618. 10.1128/AEM.02523-18. 30658982 PMC6414375

[emi470339-bib-0055] Kästner, M. , and A. Miltner . 2018. “SOM and Microbes—What Is Left From Microbial Life.” In The Future of Soil Carbon, edited by C. Garcia , P. Nannipieri , and T. Hernandez , 125–163. Academic Press. 10.1016/B978-0-12-811687-6.00005-5.

[emi470339-bib-0056] Labrie, G. , A.‐È. Gagnon , A. Vanasse , A. Latraverse , and G. Tremblay . 2020. “Impacts of Neonicotinoid Seed Treatments on Soil‐Dwelling Pest Populations and Agronomic Parameters in Corn and Soybean in Quebec (Canada).” PLoS One 15, no. 2: e0229136. 10.1371/journal.pone.0229136. 32101547 PMC7043745

[emi470339-bib-0057] Lamers, M. , M. Anyusheva , N. La , V. V. Nguyen , and T. Streck . 2011. “Pesticide Pollution in Surface‐ and Groundwater by Paddy Rice Cultivation: A Case Study From Northern Vietnam.” Clean: Soil, Air, Water 39, no. 4: 356–361. 10.1002/clen.201000268.

[emi470339-bib-0058] Lane, D. J. 1991. “16S/23S rRNA Sequencing.” In Nucleic Acid Techniques in Bacterial Systematics, edited by E. Stackebrandt and M. Goodfellow , 115–147. John Wiley & Sons.

[emi470339-bib-0059] Lane, D. J. , B. Pace , G. J. Olsen , D. A. Stahl , M. L. Sogin , and N. R. Pace . 1985. “Rapid Determination of 16S Ribosomal RNA Sequences for Phylogenetic Analyses.” Proceedings of the National Academy of Sciences 82, no. 20: 6955–6959. 10.1073/pnas.82.20.6955. PMC391288 2413450

[emi470339-bib-0060] Laranjo, M. , A. Alexandre , and S. Oliveira . 2014. “Legume Growth‐Promoting Rhizobia: An Overview on the *Mesorhizobium* Genus.” Microbiological Research 169, no. 1: 2–17. 10.1016/j.micres.2013.09.012. 24157054

[emi470339-bib-0061] Lenth, R. V. 2024. “emmeans: Estimated Marginal Means, Aka Least‐Squares Means.” 10.32614/CRAN.package.emmeans.

[emi470339-bib-0062] Li, Y. , J. An , Z. Dang , H. Lv , W. Pan , and Z. Gao . 2018. “Treating Wheat Seeds With Neonicotinoid Insecticides Does Not Harm the Rhizosphere Microbial Community.” PLoS One 13, no. 12: e0205200. 10.1371/journal.pone.0205200. 30507964 PMC6277090

[emi470339-bib-0063] Liu, S. , Z. Zheng , F. Wei , et al. 2010. “Simultaneous Determination of Seven Neonicotinoid Pesticide Residues in Food by Ultraperformance Liquid Chromatography Tandem Mass Spectrometry.” Journal of Agricultural and Food Chemistry 58, no. 6: 3271–3278. 10.1021/jf904045j. 20187609

[emi470339-bib-0064] Liu, Z. , J. Zhuang , K. Zheng , and C. Luo . 2023. “Differential Response of the Soil Nutrients, Soil Bacterial Community Structure and Metabolic Functions to Different Risk Areas in Lead‐Zine Tailings.” Frontiers in Microbiology 14: 1131770. 10.3389/fmicb.2023.1131770. 37779699 PMC10536257

[emi470339-bib-0065] Lladó, S. , S. Covino , A. M. Solanas , M. Petruccioli , A. D'annibale , and M. Viñas . 2015. “Pyrosequencing Reveals the Effect of Mobilizing Agents and Lignocellulosic Substrate Amendment on Microbial Community Composition in a Real Industrial PAH‐Polluted Soil.” Journal of Hazardous Materials 283: 35–43. 10.1016/j.jhazmat.2014.08.065. 25261758

[emi470339-bib-0066] Ma, Q. , H. Tan , J. Song , et al. 2022. “Effects of Long‐Term Exposure to the Herbicide Nicosulfuron on the Bacterial Community Structure in a Factory Field.” Environmental Pollution 307: 119477. 10.1016/j.envpol.2022.119477. 35598816

[emi470339-bib-0067] Mahapatra, B. , T. Adak , N. K. B. Patil , et al. 2017. “Imidacloprid Application Changes Microbial Dynamics and Enzymes in Rice Soil.” Ecotoxicology and Environmental Safety 144: 123–130. 10.1016/j.ecoenv.2017.06.013. 28605646

[emi470339-bib-0068] Manda, R. R. , V. A. Addanki , A. Giabardo , et al. 2023. “Soil Health Management and Microorganisms: Recent Development.” In Detection, Diagnosis and Management of Soil‐Borne Phytopathogens, edited by U. B. Singh , R. Kumar , and H. B. Singh , 437–493. Springer Nature. 10.1007/978-981-19-8307-8_18.

[emi470339-bib-0069] Martin, M. 2011. “Cutadapt Removes Adapter Sequences From High‐Throughput Sequencing Reads.” EMBnet.Journal 17, no. 1: 10–12. 10.14806/ej.17.1.200.

[emi470339-bib-0070] Martyniuk, S. , D. Pikuła , and M. Kozieł . 2019. “Soil Properties and Productivity in Two Long‐Term Crop Rotations Differing With Respect to Organic Matter Management on an Albic Luvisol.” Scientific Reports 9, no. 1: 1878. 10.1038/s41598-018-37087-4. 30755625 PMC6372592

[emi470339-bib-0071] Mawang, C.‐I. , A.‐S. Azman , A.‐S. M. Fuad , and M. Ahamad . 2021. “Actinobacteria: An Eco‐Friendly and Promising Technology for the Bioaugmentation of Contaminants.” Biotechnology Reports 32: e00679. 10.1016/j.btre.2021.e00679. 34660214 PMC8503819

[emi470339-bib-0072] Mclaren, M. R. , and B. J. Callahan . 2021. “Silva 138.1 Prokaryotic SSU Taxonomic Training Data Formatted for DADA2.” In: Zenodo (ed.).

[emi470339-bib-0073] Mcmurdie, P. J. , and S. Holmes . 2013. “Phyloseq: An R Package for Reproducible Interactive Analysis and Graphics of Microbiome Census Data.” PLoS One 8, no. 4: e61217. 10.1371/journal.pone.0061217. 23630581 PMC3632530

[emi470339-bib-0074] Meena, R. S. , S. Kumar , R. Datta , et al. 2020. “Impact of Agrochemicals on Soil Microbiota and Management: A Review.” Land 9, no. 2: 34. 10.3390/land9020034.

[emi470339-bib-0075] Meng, S. , X. Liang , T. Peng , et al. 2023. “Ecological Distribution and Function of Comammox *Nitrospira* in the Environment.” Applied Microbiology and Biotechnology 107, no. 12: 3877–3886. 10.1007/s00253-023-12557-6. 37195422

[emi470339-bib-0076] Miao, J. , Z.‐B. Du , Y.‐Q. Wu , et al. 2014. “Sub‐Lethal Effects of Four Neonicotinoid Seed Treatments on the Demography and Feeding Behaviour of the Wheat Aphid *Sitobion avenae* .” Pest Management Science 70, no. 1: 55–59. 10.1002/ps.3523. 23457039

[emi470339-bib-0077] Ni, B. , L. Xiao , D. Lin , et al. 2025. “Increasing Pesticide Diversity Impairs Soil Microbial Functions.” Proceedings of the National Academy of Sciences of the United States of America 122, no. 2: e2419917122. 10.1073/pnas.2419917122. 39786931 PMC11745395

[emi470339-bib-0078] Oksanen, J. , G. L. Simpson , F. G. Blanchet , et al. 2024. “Vegan: Community Ecology Package.” https://CRAN.R‐project.org/package=vegan.

[emi470339-bib-0079] Orikpete, O. F. , K. N. Kikanme , T. D. O. Falade , N. M. Dennis , D. R. Ejike Ewim , and O. O. Fadare . 2025. “Neonicotinoid Pesticides in African Agriculture: What Do We Know and What Should Be the Focus for Future Research?” Chemosphere 372: 144057. 10.1016/j.chemosphere.2024.144057. 39746486

[emi470339-bib-0080] Overton, K. , A. A. Hoffmann , O. L. Reynolds , and P. A. Umina . 2021. “Toxicity of Insecticides and Miticides to Natural Enemies in Australian Grains: A Review.” Insects 12, no. 2: 187. 10.3390/insects12020187. 33671702 PMC7927080

[emi470339-bib-0081] Pagès, H. , P. Aboyoun , R. Gentleman , and S. Debroy . 2024. “Biostrings: Efficient Manipulation of Biological Strings.” 10.18129/B9.bioc.Biostrings.

[emi470339-bib-0082] Paradis, E. , and K. Schliep . 2019. “Ape 5.0: An Environment for Modern Phylogenetics and Evolutionary Analyses in R.” Bioinformatics 35, no. 3: 526–528. 10.1093/bioinformatics/bty633. 30016406

[emi470339-bib-0083] Parizadeh, M. , B. Mimee , and S. W. Kembel . 2021. “Neonicotinoid Seed Treatments Have Significant Non‐Target Effects on Phyllosphere and Soil Bacterial Communities.” Frontiers in Microbiology 11: 619827. 10.3389/fmicb.2020.619827. 33584586 PMC7873852

[emi470339-bib-0084] Parizadeh, M. , B. Mimee , and S. W. Kembel . 2023. “Soil Microbial Gene Expression in an Agricultural Ecosystem Varies With Time and Neonicotinoid Seed Treatments.” Microbiology (Reading) 169, no. 4: 001318. 10.1099/mic.0.001318. 37083497 PMC10202318

[emi470339-bib-0085] Pertile, M. , R. M. S. Sousa , L. W. Mendes , et al. 2021. “Response of Soil Bacterial Communities to the Application of the Herbicides Imazethapyr and Flumyzin.” European Journal of Soil Biology 102: 103252. 10.1016/j.ejsobi.2020.103252.

[emi470339-bib-0086] Potts, J. , R. W. Brown , D. L. Jones , and P. Cross . 2023. “Seasonal Variation Is a Bigger Driver of Soil Faunal and Microbial Community Composition Than Exposure to the Neonicotinoid Acetamiprid Within *Brassica napus* Production Systems.” Soil Biology and Biochemistry 184: 109088. 10.1016/j.soilbio.2023.109088.

[emi470339-bib-0087] Qiu, D. , M. Ke , Q. Zhang , et al. 2022. “Response of Microbial Antibiotic Resistance to Pesticides: An Emerging Health Threat.” Science of the Total Environment 850: 158057. 10.1016/j.scitotenv.2022.158057. 35977623

[emi470339-bib-0088] R Core Team . 2024. “R: A Language and Environment for Statistical Computing.” https://www.R‐project.org/.

[emi470339-bib-0089] Ramette, A. 2007. “Multivariate Analyses in Microbial Ecology.” FEMS Microbiology Ecology 62, no. 2: 142–160. 10.1111/j.1574-6941.2007.00375.x. 17892477 PMC2121141

[emi470339-bib-0090] Rasmussen, A. N. , B. B. Tolar , J. R. Bargar , K. Boye , and C. A. Francis . 2024. “Diverse and Unconventional Methanogens, Methanotrophs, and Methylotrophs in Metagenome‐Assembled Genomes From Subsurface Sediments of the Slate River Floodplain, Crested Butte, CO, USA.” mSystems 9, no. 7: e00314‐24. 10.1128/msystems.00314-24. 38940520 PMC11264602

[emi470339-bib-0091] Rawal, A. , S. Lüpold , M. M. Gossner , and W. U. Blanckenhorn . 2025. “Neonicotinoids Negatively Affect Life‐History Traits in Widespread Dung Fly Species.” Environmental Pollution 383: 126763. 10.1016/j.envpol.2025.126763. 40614955

[emi470339-bib-0092] Raza, T. , M. Abbas , Amna , et al. 2023. “Impact of Silicon on Plant Nutrition and Significance of Silicon Mobilizing Bacteria in Agronomic Practices.” SILICON 15, no. 9: 3797–3817. 10.1007/s12633-023-02302-z.

[emi470339-bib-0093] Ribeiro, I. D. A. , C. G. Volpiano , L. K. Vargas , C. E. Granada , B. B. Lisboa , and L. M. P. Passaglia . 2020. “Use of Mineral Weathering Bacteria to Enhance Nutrient Availability in Crops: A Review.” Frontiers in Plant Science 11: 590774. 10.3389/fpls.2020.590774. 33362817 PMC7759553

[emi470339-bib-0094] Schmidt, S. K. , E. K. Costello , D. R. Nemergut , et al. 2007. “Biogeochemical Consequences of Rapid Microbial Turnover and Seasonal Succession in Soil.” Ecology 88, no. 6: 1379–1385. 10.1890/06-0164. 17601130

[emi470339-bib-0095] Seabaugh, J. A. , and D. M. Anderson . 2024. “Pathogenicity and Virulence of *Yersinia* .” Virulence 15, no. 1: 2316439. 10.1080/21505594.2024.2316439. 38389313 PMC10896167

[emi470339-bib-0096] Sennett, L. B. , C. Goyer , D. L. Burton , B. J. Zebarth , and S. Whitney . 2022. “Chemical Fumigation and Biofumigation Alter Soil Bacterial Community Diversity and Composition.” FEMS Microbiology Ecology 98, no. 4: fiac026. 10.1093/femsec/fiac026. 35441686

[emi470339-bib-0097] Shade, A. , H. Peter , S. D. Allison , et al. 2012. “Fundamentals of Microbial Community Resistance and Resilience.” Frontiers in Microbiology 3: 417. 10.3389/fmicb.2012.00417. 23267351 PMC3525951

[emi470339-bib-0098] Shahid, M. , M. S. Khan , and U. B. Singh . 2023. “Pesticide‐Tolerant Microbial Consortia: Potential Candidates for Remediation/Clean‐Up of Pesticide‐Contaminated Agricultural Soil.” Environmental Research 236: 116724. 10.1016/j.envres.2023.116724. 37500042

[emi470339-bib-0099] Shen, X. , B. Wang , N. Yang , et al. 2021. “ *Lysobacter enzymogenes* Antagonizes Soilborne Bacteria Using the Type IV Secretion System.” Environmental Microbiology 23, no. 8: 4673–4688. 10.1111/1462-2920.15662. 34227200

[emi470339-bib-0100] Sindhu, S. S. , P. Parmar , and M. Phour . 2014. “Nutrient Cycling: Potassium Solubilization by Microorganisms and Improvement of Crop Growth.” In Geomicrobiology and Biogeochemistry, edited by N. Parmar and A. Singh , 175–198. Springer Berlin Heidelberg. 10.1007/978-3-642-41837-2_10.

[emi470339-bib-0101] Skidmore, A. K. , A. Siegenthaler , T. Wang , et al. 2022. “Mapping the Relative Abundance of Soil Microbiome Biodiversity From eDNA and Remote Sensing.” Science of Remote Sensing 6: 100065. 10.1016/j.srs.2022.100065.

[emi470339-bib-0102] Spokas, K. , J. King , D. Wang , and S. Papiernik . 2007. “Effects of Soil Fumigants on Methanotrophic Activity.” Atmospheric Environment 41, no. 37: 8150–8162. 10.1016/j.atmosenv.2007.06.028.

[emi470339-bib-0103] Streletskii, R. , A. Astaykina , V. Cheptsov , A. Belov , and V. Gorbatov . 2023. “Effects of the Pesticides Benomyl, Metribuzin and Imidacloprid on Soil Microbial Communities in the Field.” Agriculture 13, no. 7: 1330. 10.3390/agriculture13071330.

[emi470339-bib-0104] Syngenta Australia . 2023. “Cruiser 350FS Seed Treatment Insecticide Label.” https://syngenta.my.salesforce.com/sfc/p/24000000Yk1o/a/3V000001KipG/s.TO4iggPjhABeDDrIPvKblZBYfo7R_9f0f4uQdKj.s.

[emi470339-bib-0105] Takahashi, Y. , H. Fujitani , Y. Hirono , et al. 2020. “Enrichment of Comammox and Nitrite‐Oxidizing *Nitrospira* From Acidic Soils.” Frontiers in Microbiology 11: 1737. 10.3389/fmicb.2020.01737. 32849373 PMC7396549

[emi470339-bib-0106] Tekeu, H. , T. Jeanne , J. D'astous‐Pagé , and R. Hogue . 2023. “Artificial Network Inference Analysis Reveals the Impact of Biostimulant on Bacterial Communities in Fumigated Soil for Potato Production Against Common Scab.” Frontiers in Soil Science 3: 1208909. 10.3389/fsoil.2023.1208909.

[emi470339-bib-0107] Thany, S. H. 2023. “Molecular Mechanism of Action of Neonicotinoid Insecticides.” International Journal of Molecular Sciences 24, no. 6: 5484. 10.3390/ijms24065484. 36982557 PMC10056306

[emi470339-bib-0108] Thompson, D. A. , H.‐J. Lehmler , D. W. Kolpin , et al. 2020. “A Critical Review on the Potential Impacts of Neonicotinoid Insecticide Use: Current Knowledge of Environmental Fate, Toxicity, and Implications for Human Health.” Environmental Science: Processes & Impacts 22, no. 6: 1315–1346. 10.1039/C9EM00586B. 32267911 PMC11755762

[emi470339-bib-0109] Trujillo, M. E. , K. Hong , and O. Genilloud . 2014. “The Family Micromonosporaceae.” In The Prokaryotes: Actinobacteria, edited by E. Rosenberg , E. F. Delong , S. Lory , E. Stackebrandt , and F. Thompson , 499–569. Springer Berlin Heidelberg. 10.1007/978-3-642-30138-4_196.

[emi470339-bib-0110] Umina, P. A. , G. Mcdonald , J. Maino , O. Edwards , and A. A. Hoffmann . 2019. “Escalating Insecticide Resistance in Australian Grain Pests: Contributing Factors, Industry Trends and Management Opportunities.” Pest Management Science 75, no. 6: 1494–1506. 10.1002/ps.5285. 30506966

[emi470339-bib-0111] Uzoh, I. M. , C. B. Okebalama , C. A. Igwe , and O. O. Babalola . 2021. “Management of Soil‐Microorganism: Interphase for Sustainable Soil Fertility Management and Enhanced Food Security.” In Food Security and Safety: African Perspectives, edited by O. O. Babalola , 475–494. Springer, Cham. 10.1007/978-3-030-50672-8_25.

[emi470339-bib-0112] Vekeman, B. , F.‐M. Kerckhof , G. Cremers , et al. 2016. “New *Methyloceanibacter* Diversity From North Sea Sediments Includes Methanotroph Containing Solely the Soluble Methane Monooxygenase.” Environmental Microbiology 18, no. 12: 4523–4536. 10.1111/1462-2920.13485. 27501305

[emi470339-bib-0113] Wang, C. , Y. Jiang , Y. Shao , et al. 2024. “The Influence and Risk Assessment of Multiple Pollutants on the Bacterial and Archaeal Communities in Agricultural Lands With Different Climates and Soil Properties.” Applied Soil Ecology 193: 105130. 10.1016/j.apsoil.2023.105130.

[emi470339-bib-0114] Wang, Y. , J. Men , T. Zheng , et al. 2023. “Impact of Pyroxasulfone on Sugarcane Rhizosphere Microbiome and Functioning During Field Degradation.” Journal of Hazardous Materials 455: 131608. 10.1016/j.jhazmat.2023.131608. 37178534

[emi470339-bib-0115] Wanjofu, E. I. , S. N. Venter , C. W. Beukes , E. T. Steenkamp , E. T. Gwata , and E. K. Muema . 2022. “Nodulation and Growth Promotion of Chickpea by *Mesorhizobium* Isolates From Diverse Sources.” Microorganisms 10, no. 12: 2467. 10.3390/microorganisms10122467. 36557720 PMC9783758

[emi470339-bib-0116] Wei, L. , Y. Wang , N. Li , N. Zhao , and S. Xu . 2024. “Bacteria‐Like *Gaiella* Accelerate Soil Carbon Loss by Decomposing Organic Matter of Grazing Soils in Alpine Meadows on the Qinghai–Tibet Plateau.” Microbial Ecology 87, no. 1: 104. 10.1007/s00248-024-02414-y. 39110233 PMC11306262

[emi470339-bib-0117] Wettstein, F. E. , R. Kasteel , M. F. Garcia Delgado , et al. 2016. “Leaching of the Neonicotinoids Thiamethoxam and Imidacloprid From Sugar Beet Seed Dressings to Subsurface Tile Drains.” Journal of Agricultural and Food Chemistry 64, no. 33: 6407–6415. 10.1021/acs.jafc.6b02619. 27529118

[emi470339-bib-0118] Wickham, H. 2016. ggplot2: Elegant Graphics for Data Analysis. Springer Cham. 10.1007/978-3-319-24277-4.

[emi470339-bib-0119] Wickham, H. , R. François , L. Henry , K. Müller , and D. Vaughan . 2023. “dplyr: A Grammar of Data Manipulation.” https://CRAN.R‐project.org/package=dplyr.

[emi470339-bib-0120] Wu, C. , Z. Wang , Y. Ma , et al. 2021. “Influence of the Neonicotinoid Insecticide Thiamethoxam on Soil Bacterial Community Composition and Metabolic Function.” Journal of Hazardous Materials 405: 124275. 10.1016/j.jhazmat.2020.124275. 33092881

[emi470339-bib-0121] Wu, H.‐M. 2022. “QIAGEN DNeasy PowerSoil Pro.” *protocols.io*. 10.17504/protocols.io.bp2l69411lqe/v1.

[emi470339-bib-0122] Yadav, A. N. , D. Kour , T. Kaur , et al. 2021. “Biodiversity, and Biotechnological Contribution of Beneficial Soil Microbiomes for Nutrient Cycling, Plant Growth Improvement and Nutrient Uptake.” Biocatalysis and Agricultural Biotechnology 33: 102009. 10.1016/j.bcab.2021.102009.

[emi470339-bib-0123] Yamaguchi, T. , A. Mahmood , T. Ito , and R. Kataoka . 2021. “Non‐Target Impact of Dinotefuran and Azoxystrobin on Soil Bacterial Community and Nitrification.” Bulletin of Environmental Contamination and Toxicology 106, no. 6: 996–1002. 10.1007/s00128-021-03163-1. 33687536

[emi470339-bib-0124] Yang, T. , N. Lupwayi , S.‐A. Marc , K. H. M. Siddique , and L. D. Bainard . 2021. “Anthropogenic Drivers of Soil Microbial Communities and Impacts on Soil Biological Functions in Agroecosystems.” Global Ecology and Conservation 27: e01521. 10.1016/j.gecco.2021.e01521.

[emi470339-bib-0125] Yu, B. , Z. Chen , X. Lu , et al. 2020. “Effects on Soil Microbial Community After Exposure to Neonicotinoid Insecticides Thiamethoxam and Dinotefuran.” Science of the Total Environment 725: 138328. 10.1016/j.scitotenv.2020.138328. 32294586

[emi470339-bib-0126] Zhan, Y. , N. Yan , X. Miao , Q. Li , and C. Chen . 2021. “Different Responses of Soil Environmental Factors, Soil Bacterial Community, and Root Performance to Reductive Soil Disinfestation and Soil Fumigant Chloropicrin.” Frontiers in Microbiology 12: 796191. 10.3389/fmicb.2021.796191. 34975820 PMC8714892

[emi470339-bib-0127] Zhang, C. , X. Wang , P. Kaur , and J. Gan . 2023. “A Critical Review on the Accumulation of Neonicotinoid Insecticides in Pollen and Nectar: Influencing Factors and Implications for Pollinator Exposure.” Science of the Total Environment 899: 165670. 10.1016/j.scitotenv.2023.165670. 37478949

[emi470339-bib-0128] Zhang, D. , X. Ji , Z. Meng , W. Qi , and K. Qiao . 2019. “Effects of Fumigation With 1,3‐Dichloropropene on Soil Enzyme Activities and Microbial Communities in Continuous‐Cropping Soil.” Ecotoxicology and Environmental Safety 169: 730–736. 10.1016/j.ecoenv.2018.11.071. 30502523

[emi470339-bib-0129] Zheng, T. , J. Zhang , C. Tang , Y. Zhang , and J. Duan . 2022. “Persistence and Vertical Distribution of Neonicotinoids in Soils Under Different Citrus Orchards Chrono Sequences From Southern China.” Chemosphere 286: 131584. 10.1016/j.chemosphere.2021.131584. 34293560

[emi470339-bib-0130] Zheng, Y. , M. Maruoka , K. Nanatani , et al. 2021. “High Cellulolytic Potential of the Ktedonobacteria Lineage Revealed by Genome‐Wide Analysis of CAZymes.” Journal of Bioscience and Bioengineering 131, no. 6: 622–630. 10.1016/j.jbiosc.2021.01.008. 33676867

