# Supplementary material for: Bacterial Resilience and Vulnerability to Neonicotinoid Seed Treatments in Soil: Short‐Term Community Responses
Source: Environ Microbiol Rep. 2026 Apr 7;18(2):e70339. doi: 10.1111/1758-2229.70339 (PMC13056401; doi:10.1111/1758-2229.70339)
Supplement: Supplementary file 1 — Table S1: Summary of physicochemical properties of the soil samples analysed in this study. Table S2:. Primer sequences used for amplification of the 16S rRNA gene (V1–V3 region). Table S3:. Sequencing depths by treatments. Table S4:. Bacterial relative abundance (%) at the phylum level across different treatments over time. Table S5:. Statistical test of different bacterial phyla across different treatments and time, with effect direction assessed using median and negative significance highlighted by the red circle and positive significance highlighted by the green circle. Table S6:. PERMANOVA results showing the influence of different neonicotinoid treatments, sampling day, and their interaction on soil bacterial community structure measured by Bray–Curtis dissimilarity matrix. Figure S1:. Rarefaction curves showing sequencing depth for each sample. Figure S2:. Alpha diversity indices for bacteria, measured by (a) Shannon index, (b) Simpson index. Points represent estimated marginal means (EMMs) ± 95% confidence intervals for each treatment group (Control, Imidacloprid, Thiamethoxam, and Clothianidin). Asterisks indicate significant differences among treatments within each day based on Generalised Linear Model analysis (‘**’ ≤ 0.01, ‘*’ ≤ 0.05). Figure S3:. Alpha diversity indices for bacteria, evaluated through (a) Chao1 index, (b) ACE index. Points represent estimated marginal means (EMMs) ±95% confidence intervals for each treatment group (Control, Imidacloprid, Thiamethoxam and Clothianidin). Asterisks indicate significant differences among treatments within each day based on Generalised Linear Model analysis (‘*’ ≤ 0.05). Figure S4:. Scatter plot showing CLR‐transformed relative abundance of bacterial families that showed significant Spearman correlations (p ≤ 0.05) with sampling day and/or neonicotinoid treatments. [file EMI4-18-e70339-s001.docx]

**Bacterial resilience and vulnerability to neonicotinoid seed treatments in soil: Short-term community responses**

Sharmin Akter*^1, 2^, Julia Jasonsmith^1^, Nilantha R. Hulugalle^1^, Craig L. Strong^1^

^1^Fenner School of Environment and Society, College of Systems and Society, Australian National University, Canberra, ACT, Australia.

^2^Soil Resource Development Institute, Ministry of Agriculture, Dhaka, Bangladesh.

*** Corresponding author:**

Sharmin Akter, Fenner School of Environment and Society, Australian National University, Australia.

Email: [sharmin.akter@anu.edu.au](mailto:sharmin.akter@anu.edu.au)

ORCID: 0000-0002-4086-3439

**List of Supplementary tables:**

**Table S1**. Summary of physicochemical properties of the soil samples analysed in this study

**Table S2**. Primer sequences used for amplification of the 16S rRNA gene (V1–V3 region)

**Table S3**. Sequencing depths by treatments

**Table S4**. Bacterial relative abundance (%) at the phylum level across different treatments over time

**Table S5**. Statistical test of different bacterial phyla across different treatments and time, with effect direction assessed using median and negative significance highlighted by the red circle and positive significance highlighted by the green circle

**Table S6**. PERMANOVA results showing the influence of different neonicotinoid treatments, sampling day, and their interaction on soil bacterial community structure measured by Bray-Curtis dissimilarity matrix

**List of Supplementary figures:**

**Figure S1**. Rarefaction curves showing sequencing depth for each sample.

**Figure S2**. Alpha diversity indices for bacteria, measured by a) Shannon index, b) Simpson index. Points represent estimated marginal means (EMMs) ± 95% confidence intervals for each treatment group (Control, Imidacloprid, Thiamethoxam, and Clothianidin). Asterisks indicate significant differences among treatments within each day based on Generalised Linear Model analysis (‘**’ ≤ 0.01, ‘*’ ≤ 0.05).

**Figure S3**. Alpha diversity indices for bacteria, evaluated through a) Chao1 index, b) ACE index. Points represent estimated marginal means (EMMs) ± 95% confidence intervals for each treatment group (Control, Imidacloprid, Thiamethoxam, and Clothianidin). Asterisks indicate significant differences among treatments within each day based on Generalised Linear Model analysis (‘*’ ≤ 0.05).

**Figure S4**. Scatter plot showing CLR-transformed relative abundance of bacterial families that showed significant Spearman correlations (*P* ≤ 0.05) with sampling day and/or neonicotinoid treatments.

Table S1. Summary of physicochemical properties of the soil samples analysed in this study

| Parameter | Method | Unit | Method reference |
| --- | --- | --- | --- |
| Texture | USDA textural triangle | - | Soil Survey Division Staff (1993) |
| Clay | Hydrometer method | g/100 g | (modified) Carter and Gregorich (2007) |
| Silt | Hydrometer method | g/100 g |  |
| Sand | Hydrometer method | g/100 g |  |
| pH | 1:5 soil:water suspension | - | Rayment and Lyons (2011) |
| Electrical conductivity (EC) | 1:5 soil:water extract | dS/m |  |
| Organic matter | Calculation: Total Carbon × 1.75 | g/100 g |  |
| Cation exchange capacity (CEC) | A sum of Ca, Mg, K, Na | - |  |
| Exchangeable Ca | Ammonium Acetate (1M NH_4_OAc) | cmol_+_/kg |  |
| Exchangeable Mg |  | cmol_+_/kg |  |
| Exchangeable Ka |  | cmol_+_/kg |  |
| Exchangeable Na |  | cmol_+_/kg |  |
| Ca/ Mg ratio | Calculation: Ca/ Mg | - |  |
| Phosphorus | Fluoride-extractable P (Bray 1-P) | mg/kg |  |
| Total Carbon | LECO TruMac Analyzer | g/100 g | LECO Corporation (2015) |
| Total Nitrogen | LECO TruMac Analyzer | g/100 g |  |
| C/ N ratio | Calculation:  Total Carbon/ Total Nitrogen | - |  |

Table S2. Primer sequences used for amplification of the 16S rRNA gene (V1–V3 region)

| Primer name | Component | Primer orientation | Sequence (5'–3') | Reference |
| --- | --- | --- | --- | --- |
| 27f | Illumina forward overhang | Forward | TCGTCGGCAGCGTCAGATGTGTATAAGAGACAG | - |
|  | Locus-specific primer |  | AGAGTTTGATCMTGGCTCAG | Lane (1991) |
| 519r | Illumina reverse overhang | Reverse | GTCTCGTGGGCTCGGAGATGTGTATAAGAGACAG | - |
|  | Locus-specific primer |  | GWATTACCGCGGCKGCTG | Lane et al. (1985) |

Table S3. Sequencing depths by treatments

| Treatment | No. of samples | Mean reads (± SD) | Median reads | Minimum reads | Maximum reads |
| --- | --- | --- | --- | --- | --- |
| Control | 30 | 5718 ± 2212 | 6132 | 391 | 9657 |
| Imidacloprid | 30 | 6313 ± 2301 | 6752 | 1881 | 11209 |
| Thiamethoxam | 30 | 6102 ± 1975 | 6626 | 728 | 9861 |
| Clothianidin | 30 | 6328 ± 2696 | 5572 | 2052 | 11565 |

Table S4. Bacterial relative abundance (%) at the phylum level across different treatments over time

| Treatment | Relative abundance (%) | | | | | | | | | | | | | | | | | | | | | | | |
| --- | --- | --- | --- | --- | --- | --- | --- | --- | --- | --- | --- | --- | --- | --- | --- | --- | --- | --- | --- | --- | --- | --- | --- | --- |
|  | Control | | | | | | Imidacloprid | | | | | | Thiamethoxam | | | | | | Clothianidin | | | | | |
| Day | 1 | 2 | 3 | 5 | 7 | 10 | 1 | 2 | 3 | 5 | 7 | 10 | 1 | 2 | 3 | 5 | 7 | 10 | 1 | 2 | 3 | 5 | 7 | 10 |
| Phylum | | | | | | | | | | | | | | | | | | | | | | | | |
| Abditibacteriota | 0.00 | 0.01 | 0.00 | 0.02 | 0.01 | 0.02 | 0.02 | 0.01 | 0.01 | 0.01 | 0.00 | 0.00 | 0.00 | 0.00 | 0.01 | 0.01 | 0.00 | 0.03 | 0.00 | 0.01 | 0.00 | 0.02 | 0.01 | 0.01 |
| Acidobacteriota | 3.51 | 3.18 | 3.13 | 3.54 | 3.74 | 4.00 | 3.57 | 3.63 | 3.80 | 3.22 | 3.74 | 3.41 | 3.75 | 3.51 | 3.45 | 3.72 | 4.03 | 2.79 | 3.91 | 3.18 | 3.79 | 3.64 | 3.82 | 2.43 |
| Actinobacteriota | 36.92 | 39.34 | 36.13 | 37.49 | 36.82 | 38.49 | 33.55 | 38.16 | 37.07 | 36.92 | 37.35 | 34.95 | 37.42 | 37.15 | 38.55 | 37.05 | 37.57 | 37.14 | 33.55 | 39.48 | 32.22 | 37.93 | 39.02 | 44.91 |
| Armatimonadota | 0.00 | 0.01 | 0.00 | 0.00 | 0.01 | 0.01 | 0.00 | 0.00 | 0.01 | 0.00 | 0.00 | 0.01 | 0.00 | 0.00 | 0.00 | 0.00 | 0.01 | 0.00 | 0.00 | 0.00 | 0.00 | 0.00 | 0.01 | 0.01 |
| Bacteroidota | 8.98 | 9.05 | 8.47 | 8.41 | 8.61 | 7.70 | 10.05 | 8.10 | 9.29 | 8.37 | 7.80 | 7.97 | 10.10 | 8.34 | 9.16 | 8.60 | 8.19 | 7.74 | 10.95 | 7.56 | 9.93 | 9.81 | 7.42 | 5.66 |
| Bdellovibrionota | 0.14 | 0.18 | 0.19 | 0.16 | 0.24 | 0.22 | 0.21 | 0.16 | 0.21 | 0.19 | 0.24 | 0.22 | 0.16 | 0.18 | 0.11 | 0.18 | 0.15 | 0.21 | 0.15 | 0.26 | 0.25 | 0.22 | 0.19 | 0.15 |
| Chloroflexi | 2.14 | 2.44 | 1.57 | 1.86 | 1.82 | 1.76 | 2.46 | 1.54 | 2.00 | 1.63 | 1.66 | 1.49 | 2.35 | 1.83 | 1.35 | 1.77 | 1.92 | 1.48 | 1.74 | 1.50 | 1.91 | 2.06 | 1.53 | 1.27 |
| Cyanobacteria | 0.03 | 0.02 | 0.02 | 0.04 | 0.04 | 0.07 | 0.03 | 0.00 | 0.01 | 0.04 | 0.03 | 0.02 | 0.05 | 0.04 | 0.03 | 0.02 | 0.06 | 0.02 | 0.03 | 0.02 | 0.03 | 0.05 | 0.02 | 0.02 |
| Dependentiae | 0.26 | 0.21 | 0.11 | 0.23 | 0.38 | 0.27 | 0.20 | 0.15 | 0.22 | 0.21 | 0.25 | 0.25 | 0.51 | 0.16 | 0.20 | 0.27 | 0.19 | 0.19 | 0.23 | 0.11 | 0.18 | 0.14 | 0.23 | 0.14 |
| Desulfobacterota | 0.02 | 0.01 | 0.03 | 0.00 | 0.01 | 0.01 | 0.02 | 0.00 | 0.02 | 0.00 | 0.01 | 0.00 | 0.03 | 0.01 | 0.00 | 0.01 | 0.02 | 0.04 | 0.00 | 0.00 | 0.00 | 0.00 | 0.04 | 0.02 |
| Entotheonellaeota | 0.06 | 0.04 | 0.02 | 0.00 | 0.03 | 0.01 | 0.04 | 0.08 | 0.02 | 0.04 | 0.01 | 0.02 | 0.01 | 0.03 | 0.01 | 0.03 | 0.02 | 0.01 | 0.04 | 0.04 | 0.05 | 0.02 | 0.02 | 0.00 |
| Fibrobacterota | 0.07 | 0.08 | 0.07 | 0.04 | 0.13 | 0.10 | 0.06 | 0.03 | 0.06 | 0.09 | 0.05 | 0.06 | 0.06 | 0.09 | 0.01 | 0.04 | 0.02 | 0.04 | 0.08 | 0.11 | 0.07 | 0.16 | 0.09 | 0.02 |
| Firmicutes | 7.73 | 7.11 | 7.92 | 8.61 | 8.45 | 8.20 | 8.16 | 8.21 | 8.05 | 8.99 | 8.39 | 14.09 | 7.11 | 8.47 | 8.39 | 8.57 | 8.00 | 10.85 | 7.92 | 7.98 | 8.52 | 8.22 | 8.18 | 10.42 |
| Gemmatimonadota | 1.30 | 1.46 | 1.28 | 1.23 | 1.30 | 1.23 | 1.35 | 1.39 | 1.34 | 1.21 | 1.38 | 1.09 | 1.06 | 1.75 | 1.34 | 1.55 | 1.26 | 1.23 | 1.31 | 1.33 | 1.41 | 1.24 | 1.36 | 1.49 |
| Hydrogenedentes | 0.00 | 0.02 | 0.01 | 0.01 | 0.00 | 0.00 | 0.00 | 0.00 | 0.00 | 0.00 | 0.02 | 0.02 | 0.02 | 0.01 | 0.00 | 0.02 | 0.05 | 0.02 | 0.03 | 0.01 | 0.00 | 0.02 | 0.01 | 0.00 |
| Methylomirabilota | 0.00 | 0.00 | 0.00 | 0.00 | 0.00 | 0.00 | 0.02 | 0.02 | 0.05 | 0.01 | 0.01 | 0.02 | 0.02 | 0.01 | 0.03 | 0.03 | 0.02 | 0.02 | 0.00 | 0.00 | 0.00 | 0.02 | 0.02 | 0.00 |
| Myxococcota | 4.93 | 4.39 | 4.27 | 4.32 | 4.77 | 4.77 | 5.33 | 4.87 | 4.85 | 4.75 | 4.91 | 4.32 | 4.82 | 4.91 | 4.39 | 5.35 | 4.46 | 4.20 | 4.99 | 4.62 | 4.38 | 4.41 | 5.14 | 4.04 |
| Nitrospirota | 1.00 | 1.39 | 0.63 | 1.01 | 1.09 | 1.03 | 1.03 | 0.94 | 1.05 | 0.98 | 1.01 | 0.78 | 1.20 | 0.75 | 0.84 | 0.77 | 1.23 | 0.60 | 0.86 | 0.72 | 0.86 | 0.87 | 0.98 | 0.29 |
| Patescibacteria | 0.03 | 0.04 | 0.03 | 0.02 | 0.01 | 0.01 | 0.05 | 0.03 | 0.03 | 0.04 | 0.06 | 0.06 | 0.02 | 0.04 | 0.03 | 0.04 | 0.00 | 0.07 | 0.03 | 0.04 | 0.03 | 0.01 | 0.00 | 0.03 |
| Planctomycetota | 2.12 | 2.18 | 1.41 | 1.81 | 1.91 | 1.64 | 2.01 | 1.43 | 1.97 | 1.32 | 1.59 | 1.30 | 2.21 | 1.70 | 1.39 | 1.80 | 1.52 | 1.22 | 2.36 | 1.49 | 2.01 | 1.62 | 1.43 | 1.05 |
| Proteobacteria | 28.63 | 26.39 | 32.84 | 29.27 | 28.77 | 28.71 | 29.40 | 29.59 | 27.98 | 30.21 | 29.88 | 28.40 | 27.38 | 29.06 | 29.00 | 28.16 | 29.59 | 30.53 | 29.63 | 29.58 | 32.19 | 27.35 | 28.71 | 26.03 |
| Spirochaetota | 0.02 | 0.00 | 0.00 | 0.02 | 0.00 | 0.01 | 0.00 | 0.03 | 0.03 | 0.02 | 0.01 | 0.02 | 0.01 | 0.03 | 0.01 | 0.01 | 0.01 | 0.01 | 0.02 | 0.02 | 0.02 | 0.01 | 0.01 | 0.00 |
| Sumerlaeota | 0.01 | 0.00 | 0.01 | 0.01 | 0.03 | 0.01 | 0.01 | 0.01 | 0.02 | 0.00 | 0.01 | 0.01 | 0.00 | 0.01 | 0.01 | 0.01 | 0.00 | 0.01 | 0.00 | 0.00 | 0.01 | 0.00 | 0.01 | 0.01 |
| Verrucomicrobiota | 2.08 | 2.43 | 1.86 | 1.90 | 1.84 | 1.72 | 2.43 | 1.61 | 1.94 | 1.74 | 1.60 | 1.49 | 1.69 | 1.92 | 1.68 | 1.99 | 1.69 | 1.55 | 2.16 | 1.94 | 2.12 | 2.18 | 1.75 | 2.00 |

Table S5. Statistical test of different bacterial phyla across different treatments and time, with effect direction assessed using median and negative significance highlighted by the red circle and positive significance highlighted by the green circle

| Phylum | Test type | P-value (Day) | | P-value (Treatment) | |
| --- | --- | --- | --- | --- | --- |
| Abditibacteriota | Kruskal-Wallis | 0.13 |  | 0.90 |  |
| Acidobacteriota | Kruskal-Wallis | 0.18 |  | 0.94 |  |
| Actinobacteriota | Kruskal-Wallis | 0.20 |  | 0.56 |  |
| Armatimonadota | Kruskal-Wallis | 0.10 |  | 0.94 |  |
| Bacteroidota | Kruskal-Wallis | 0.01 |  | 0.99 |  |
| Bdellovibrionota | ANOVA | 0.77 |  | 0.30 |  |
| Chloroflexi | ANOVA | 0.09 |  | 0.47 |  |
| Cyanobacteria | ANOVA | 0.51 |  | 0.17 |  |
| Dependentiae | Kruskal-Wallis | 0.19 |  | 0.22 |  |
| Desulfobacterota | Kruskal-Wallis | 0.16 |  | 0.60 |  |
| Entotheonellaeota | ANOVA | 0.11 |  | 0.62 |  |
| Fibrobacterota | ANOVA | 0.86 |  | 0.13 |  |
| Firmicutes | Kruskal-Wallis | 0.02 |  | 0.54 |  |
| Gemmatimonadota | ANOVA | 0.32 |  | 0.80 |  |
| Hydrogenedentes | Kruskal-Wallis | 0.56 |  | 0.21 |  |
| Methylomirabilota | Kruskal-Wallis | 0.92 |  | 0.001 |  |
| Myxococcota | ANOVA | 0.07 |  | 0.46 |  |
| Nitrospirota | ANOVA | 0.10 |  | 0.16 |  |
| Patescibacteria | ANOVA | 0.35 |  | 0.10 |  |
| Planctomycetota | ANOVA | 0.01 |  | 0.41 |  |
| Proteobacteria | ANOVA | 0.59 |  | 0.98 |  |
| Spirochaetota | ANOVA | 0.41 |  | 0.29 |  |
| Sumerlaeota | Kruskal-Wallis | 0.34 |  | 0.81 |  |
| Verrucomicrobiota | ANOVA | 0.11 |  | 0.10 |  |

Table S6. PERMANOVA results showing the influence of different neonicotinoid treatments, sampling day, and their interaction on soil bacterial community structure measured by Bray-Curtis dissimilarity matrix

| Source of variation | PERMANOVA | | | | |
| --- | --- | --- | --- | --- | --- |
|  | df | SS | R^2^ | F | PR (>F) |
| Day | 5 | 1.14 | 0.05 | 1.20 | 0.013* |
| Treatment | 3 | 0.54 | 0.02 | 0.95 | 0.664 |
| Day:Treatment | 15 | 3.26 | 0.14 | 1.14 | 0.004* |
| Residual | 96 | 18.23 | 0.79 | NA | NA |
| Total | 119 | 23.17 | 1.00 | NA | NA |

Signif. codes: ‘***’ ≤ 0.001 ‘**’ ≤ 0.01 ‘*’ ≤ 0.05.


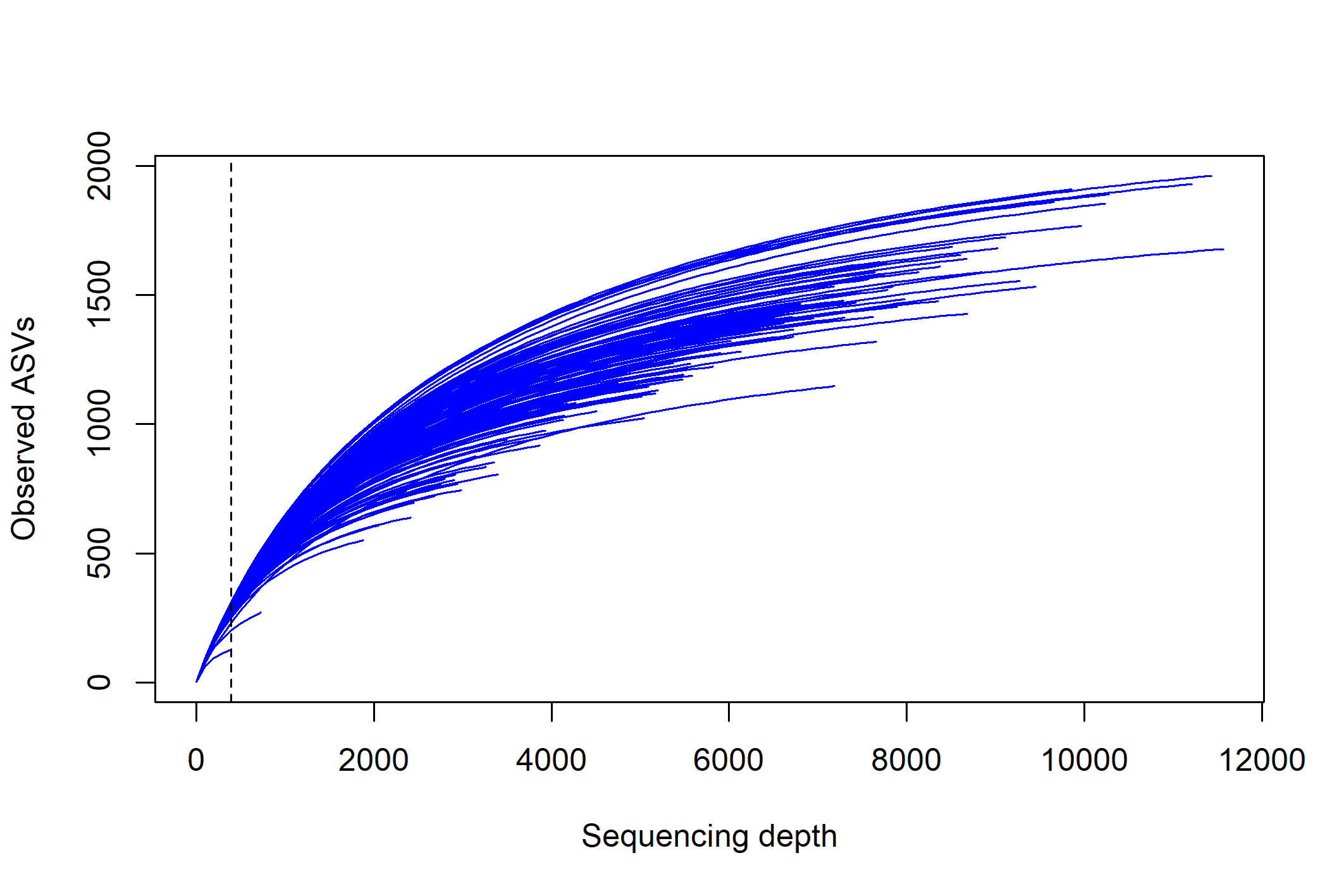


Figure S1. Barplot showing sequencing depth for each sample. Samples are not rarefied. Horizontal variation reflects natural differences in library size.


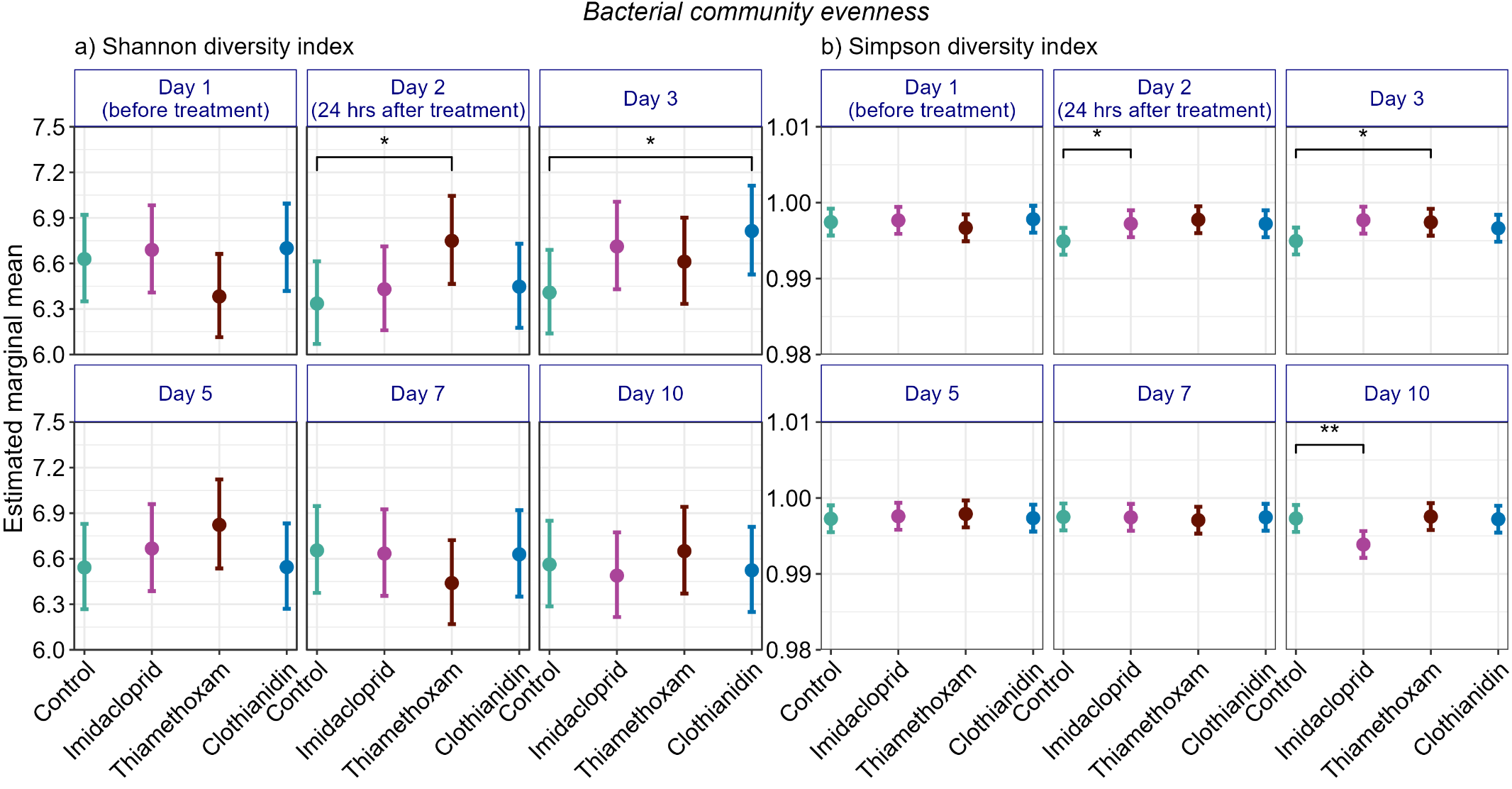


Figure S2. Alpha diversity indices for bacteria, measured by a) Shannon index, b) Simpson index. Points represent estimated marginal means (EMMs) ± 95% confidence intervals for each treatment group (Control, Imidacloprid, Thiamethoxam, and Clothianidin). Asterisks indicate significant differences among treatments within each day based on Generalised Linear Model analysis (‘**’ ≤ 0.01, ‘*’ ≤ 0.05).


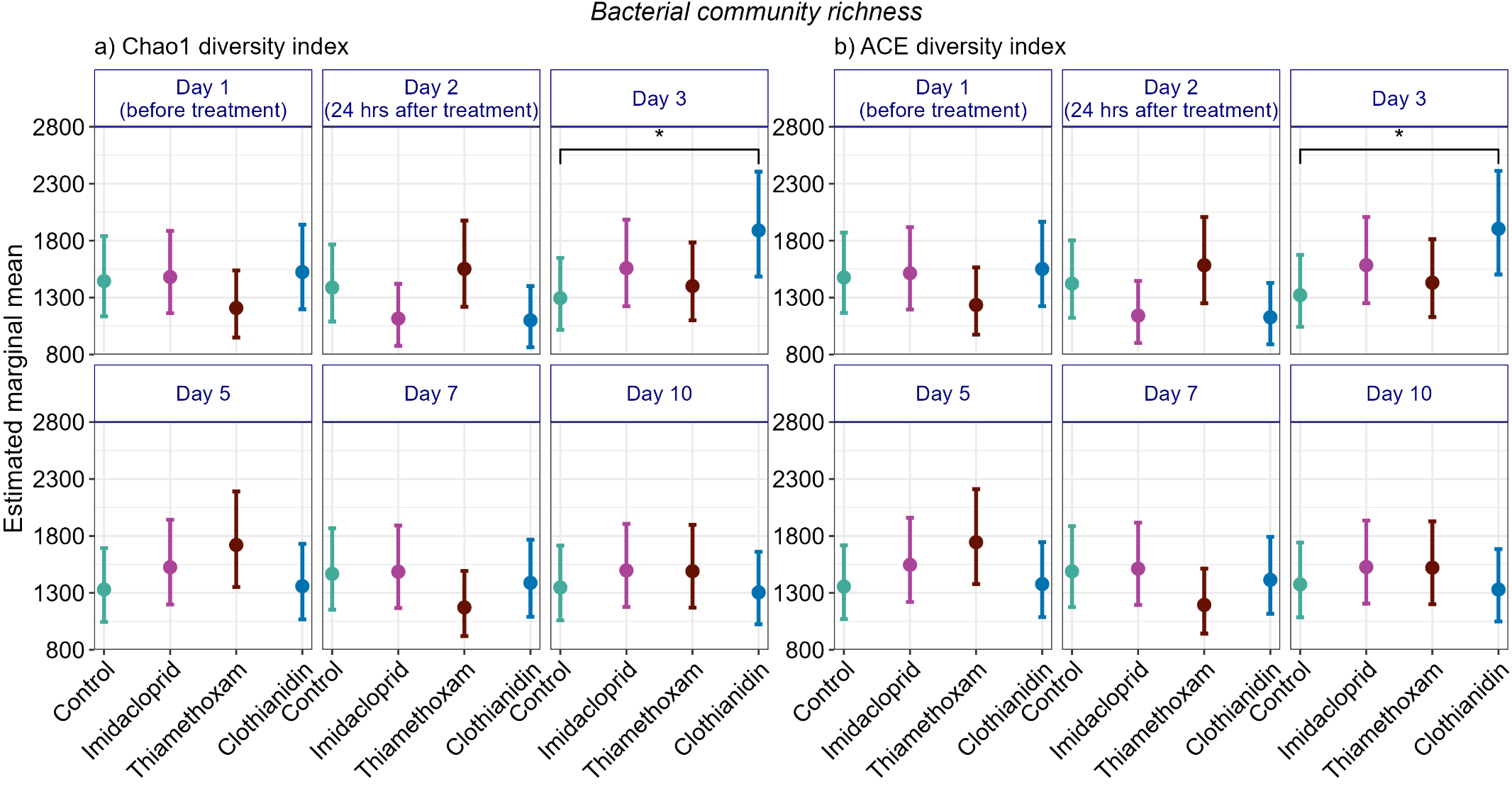


Figure S3. Alpha diversity indices for bacteria, evaluated through a) Chao1 index, b) ACE index. Points represent estimated marginal means (EMMs) ± 95% confidence intervals for each treatment group (Control, Imidacloprid, Thiamethoxam, and Clothianidin). Asterisks indicate significant differences among treatments within each day based on Generalised Linear Model analysis (‘*’ ≤ 0.05).


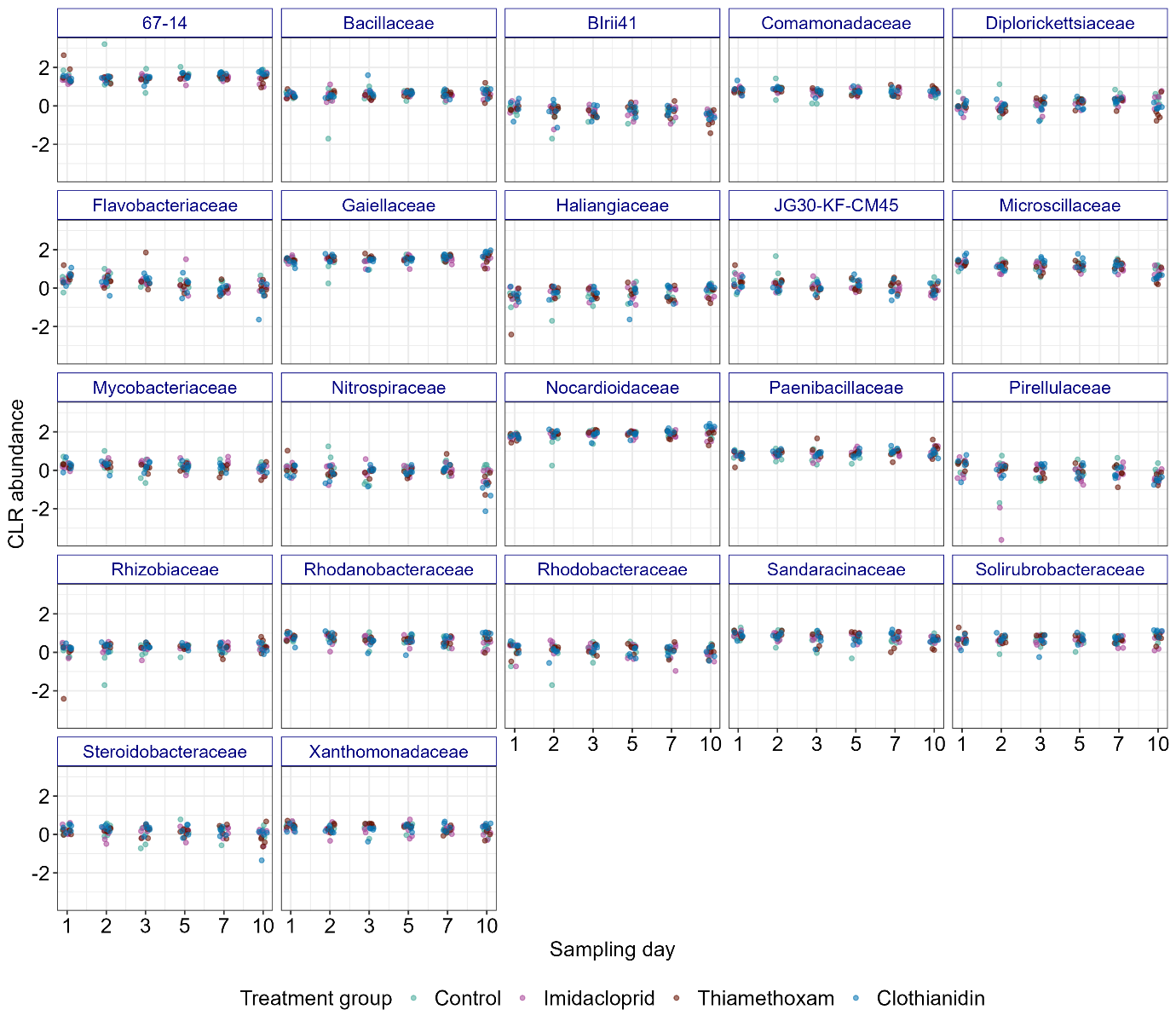


Figure S4. Scatter plot showing CLR-transformed relative abundance of bacterial families that showed significant Spearman correlations (*P* ≤ 0.05) with sampling day and/or neonicotinoid treatments.

**References**

Carter M. R. & Gregorich E. G. (2007) *Soil sampling and methods of analysis*. USA: CRC Press. <https://doi.org/10.1201/9781420005271>.

Lane, D. J. (1991) 16S/23S rRNA sequencing. In: E. Stackebrandt & G. Michael, eds. *Nucleic acid techniques in bacterial systematics*. Chichester, United Kingdom: John Wiley & Sons, pp. 115-175.

Lane, D. J., Pace, B., Olsen, G. J., Stahl, D. A., Sogin, M. L. & Pace, N. R. (1985) Rapid determination of 16S ribosomal RNA sequences for phylogenetic analyses. *Proceedings of the National Academy of Sciences*, 82, pp. 6955-6959.

LECO Corporation (2015) *Carbon, nitrogen, and sulfur in soil (Instrument: TruMac® CNS)*. Available at: <https://www.leco.com> [Accessed 14 April 2025].

Rayment, G. E. & Lyons, D. J. (2011) *Soil chemical methods - Australasia*. Collingwood, Victoria: CSIRO PUBLISHING.

Soil Survey Division Staff (1993) *Soil survey manual*. Washington DC, USA: US Department of Agriculture.
